# Elevated Apolipoprotein E Expression in Hippocampal Microglia Drives Temporal Lobe Epilepsy Progression

**DOI:** 10.1002/advs.202505778

**Published:** 2025-10-14

**Authors:** Jianwei Shi, Zesheng Li, Xin Sun, Yanfeng Yang, Yumin Luo, Ziang Song, Hengxin Dong, Lei Jin, Jing Xie, Yongzhi Shan, Guoguang Zhao

**Affiliations:** ^1^ Department of Neurosurgery Xuanwu Hospital Capital Medical University No. 45 Changchun Road Beijing 100053 China; ^2^ China International Neuroscience Institute No. 45 Changchun Road Beijing 100053 China; ^3^ Clinical Research Center for Epilepsy Capital Medical University No. 45 Changchun Road Beijing 100053 China; ^4^ Department of Pharmacology Jiangsu Key Laboratory of Neurodegeneration Nanjing Medical University No. 101 Longmian Road Nanjing 211166 China; ^5^ Deanery of Biomedical Sciences Edinburgh Medical School College of Medicine and Veterinary Medicine University of Edinburgh Edinburgh EH8 9AG UK; ^6^ Jiangsu Botanical Medicine Refinement Engineering Research Center Nanjing University of Chinese Medicine Nanjing 210023 China

**Keywords:** apolipoprotein E, metabolomics, microglia, neuroinflammation, neuronal excitability, temporal lobe epilepsy

## Abstract

Temporal lobe epilepsy (TLE), the most common form of epilepsy, is primarily characterized by hippocampal sclerosis (HS). Microglia reactivity is a critical component of TLE pathogenesis, and apolipoprotein E (APOE) may be a potential mediator of these processes. However, its role in TLE progression remains unclear. Bioinformatics approaches with biomarker validation are integrated to elucidate APOE's role and hippocampal microglia in the mechanisms underlying TLE. APOE expression is significantly elevated in the hippocampal tissues of patients with TLE‐HS and in TLE mouse models. Single‐cell RNA sequencing reveals a subset of microglia with high *APOE* gene expression, which serves as the principal carrier of increased *APOE* during disease progression. Bioinformatic analyses, in vitro studies, and in vivo functional experiments utilizing TLE mouse models implicate these *APOE*‐expressing microglia in regulating microglial differentiation, promoting neuroinflammation, neuronal apoptosis, and enhancing neuronal excitability. Genetic knockout of *APOE* mitigates gliosis, neuronal cell death, and seizure frequency in the hippocampus of epileptic mice. Additionally, *APOE* expression primarily induces significant alterations in glycerophospholipid metabolism and its associated metabolic derivatives within the epileptic microenvironment. Overall, *APOE*‐expressing microglia are pivotal drivers of HS and TLE progression, positioning APOE and its downstream signaling pathways as promising therapeutic TLE targets.

## Graphical abstract

In temporal lobe epilepsy, hippocampal APOE is markedly upregulated predominantly in microglia. *APOE* overexpression in microglia drives TLR4 and cGAS/STING‐dependent neuroinflammation, engages bidirectional crosstalk with neurons and astrocytes, increases neuronal excitability, and perturbs hippocampal lipid metabolism. These findings suggest that APOE‐expressing microglia drive HS and TLE progression, implicating APOE and downstream pathways as therapeutic targets.

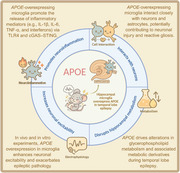

## 1 Introduction

Epilepsy affects ≈65 million individuals worldwide, with about one‐third experiencing drug‐resistant epilepsy.^[^ ^1^ ^]^ These patients often experience psychiatric and cognitive comorbidities, along with medication‐related adverse effects that significantly diminish their quality of life. Despite advancements in antiepileptic drugs targeting neuronal and synaptic mechanisms, effective seizure control remains elusive.^[^ ^2^ ^]^ Even with the introduction of over 10 third‐generation antiepileptic drugs, many patients continue to experience refractory seizures.^[^ ^3^ ^]^ Advances in the genetic understanding of epilepsy and the development of novel disease models suggest new therapeutic avenues; however, current treatments primarily focus on symptom management rather than on modifying or preventing disease progression.^[^ ^4^ ^]^ Furthermore, drug side effects often impair clinical outcomes and further reduce patients’ health‐related quality of life.

Neuroinflammation is increasingly recognized as a key driver of epileptogenesis and seizure persistence, influencing neuronal excitability and network dynamics. Within this pro‐inflammatory landscape, microglia, the resident immune cells of the central nervous system, are emerging as key players in both seizure initiation and progression. Recent studies highlight the pivotal role of microglia in epilepsy pathogenesis, particularly in shaping the neuroinflammatory milieu and modulating neuronal activity.^[^ ^5^ ^]^ These cells not only contribute to the onset and evolution of epileptic networks but may also affect seizure severity and therapeutic response.^[^ ^6^ ^]^ A deeper understanding of the intricate microglia‐neuron interplay could lead to the identification of novel therapeutic targets, ultimately improving the prognosis for patients with refractory epilepsy.

APOE (apolipoprotein E) is the principal lipid transporter in the central nervous system (CNS).^[^ ^7^ ^]^ In humans, the *APOE* gene has three allelic variants: *APOE ϵ2*, *APOE ϵ3*, and *APOE ϵ4*. The *APOE ϵ4* allele is associated with an increased risk of Alzheimer's disease, exacerbation of disability in multiple sclerosis, and a higher incidence of late‐onset post‐traumatic seizures in individuals with moderate to severe brain injuries.^[^ ^8^ ^]^ The *APOE* gene encodes the APOE protein, which is primarily produced by astrocytes in the brain.^[^ ^7^, ^9^ ^]^ Chen et al. were the first to identify reactive astrocytes with lipid accumulation (LARA) in the brains of patients with temporal lobe epilepsy (TLE).^[^ ^10^ ^]^ These LARA cells develop via an APOE‐dependent pathway and display unique molecular and functional phenotypes. Specifically, they enhance neuronal excitability by upregulating the adenosine A2A receptor and decreasing glutamate reuptake, thereby driving epilepsy progression and suggesting a new therapeutic target.^[^ ^10^ ^]^ These findings highlight the critical role of the *APOE* gene in epilepsy progression. Beyond epilepsy, APOE regulation and polymorphisms play crucial roles in neurodegenerative diseases, such as Alzheimer's and Parkinson's diseases. Recent studies suggest that the differential effects of APOE alleles on amyloid‐β accumulation and neuroinflammation are key to understanding their roles in Alzheimer's disease pathology.^[^ ^11^ ^]^ However, the specific role of APOE in epilepsy remains poorly understood, particularly regarding its involvement in microglial regulation of neuroinflammation and neuronal damage during epilepsy progression. Currently, no related reports are available.

**Figure 1:**
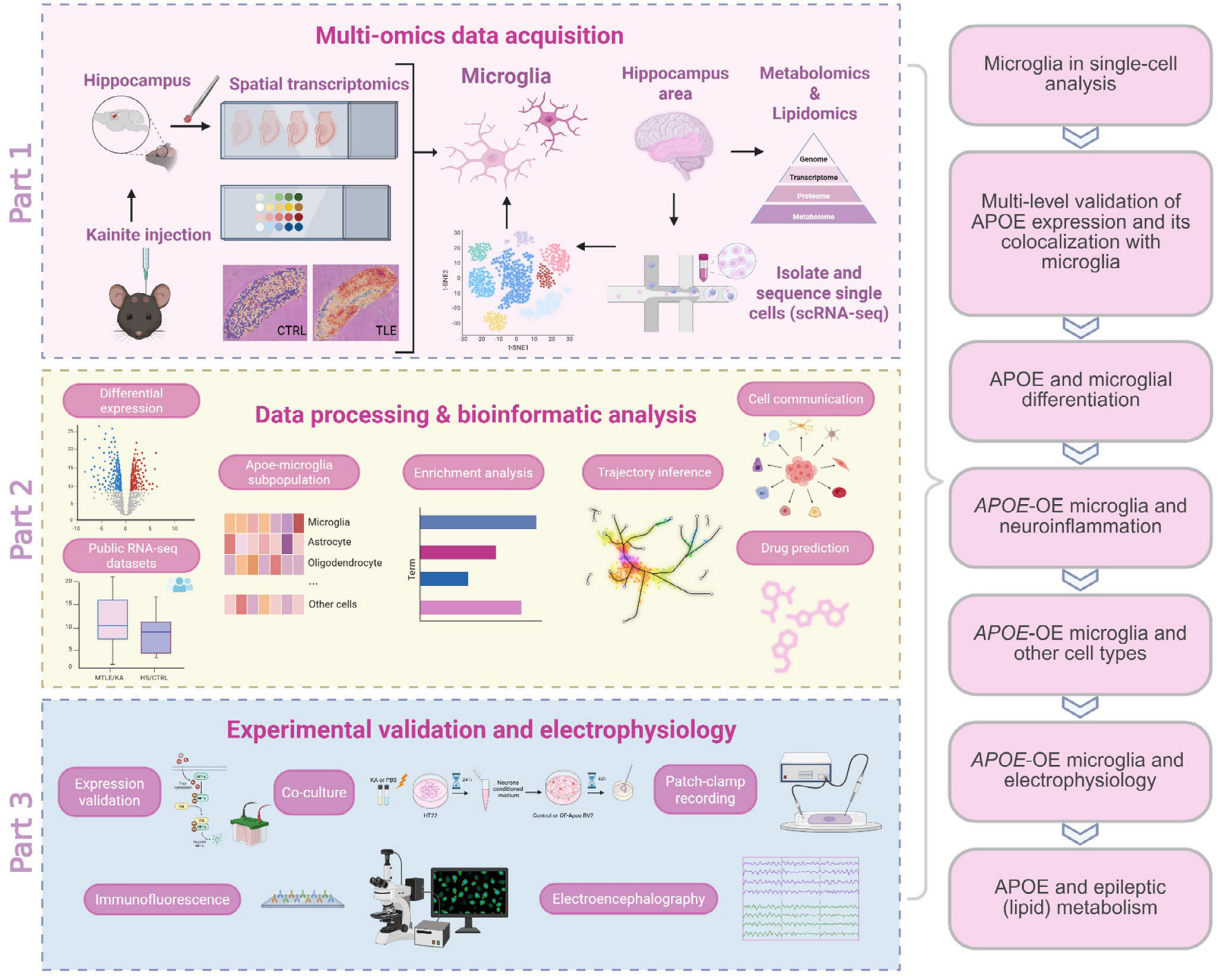
Study overview and design. Using integrated multi‐omics, including single‐cell and spatial transcriptomics, metabolomic and lipidomic profiling, together with in vivo and in vitro experiments (left), this study investigates how microglial APOE drives temporal lobe epilepsy by altering microglia differentiation, neuroinflammation, cell‐cell communication, electrophysiology, and metabolism (right). Created by BioRender.com.

Glia cell activity and neuron‐glia interactions undergo significant changes during the pre‐seizure period and seizure episodes, potentially contributing to neuronal hyperactivation and increased synchronization.^[^ ^12^ ^]^ Single‐cell transcriptomics provides insights into the functions of various cell types in epilepsy. Spatial transcriptomics and metabolomics enable researchers to analyze transcriptional changes in glial cells and neurons at the cellular level, revealing their inflammatory and metabolic profiles within the epileptic environment. These findings offer valuable insights for identifying novel therapeutic targets.^[^ ^13^ ^]^ TLE is the most common form of epilepsy, with hippocampal sclerosis (HS) as its most frequently observed pathological lesion. Multiple studies have demonstrated that neurons and glial cells exhibit marked heterogeneity across different brain regions.^[^ ^14^, ^15^, ^16^ ^]^ Because the hippocampal gyrus is often considered a “seizure gate,” we integrate bioinformatics approaches with biomarker validation to elucidate the roles of APOE and hippocampal microglia in the mechanisms underlying TLE (**Figure** **1**), thereby advancing the identification of novel therapeutic targets.

## 2 Results

### 2.1 Single‐Cell RNA Sequencing of Hippocampal Cell Suspensions from Temporal Lobe Epileptic Mice

**Figure 2:**
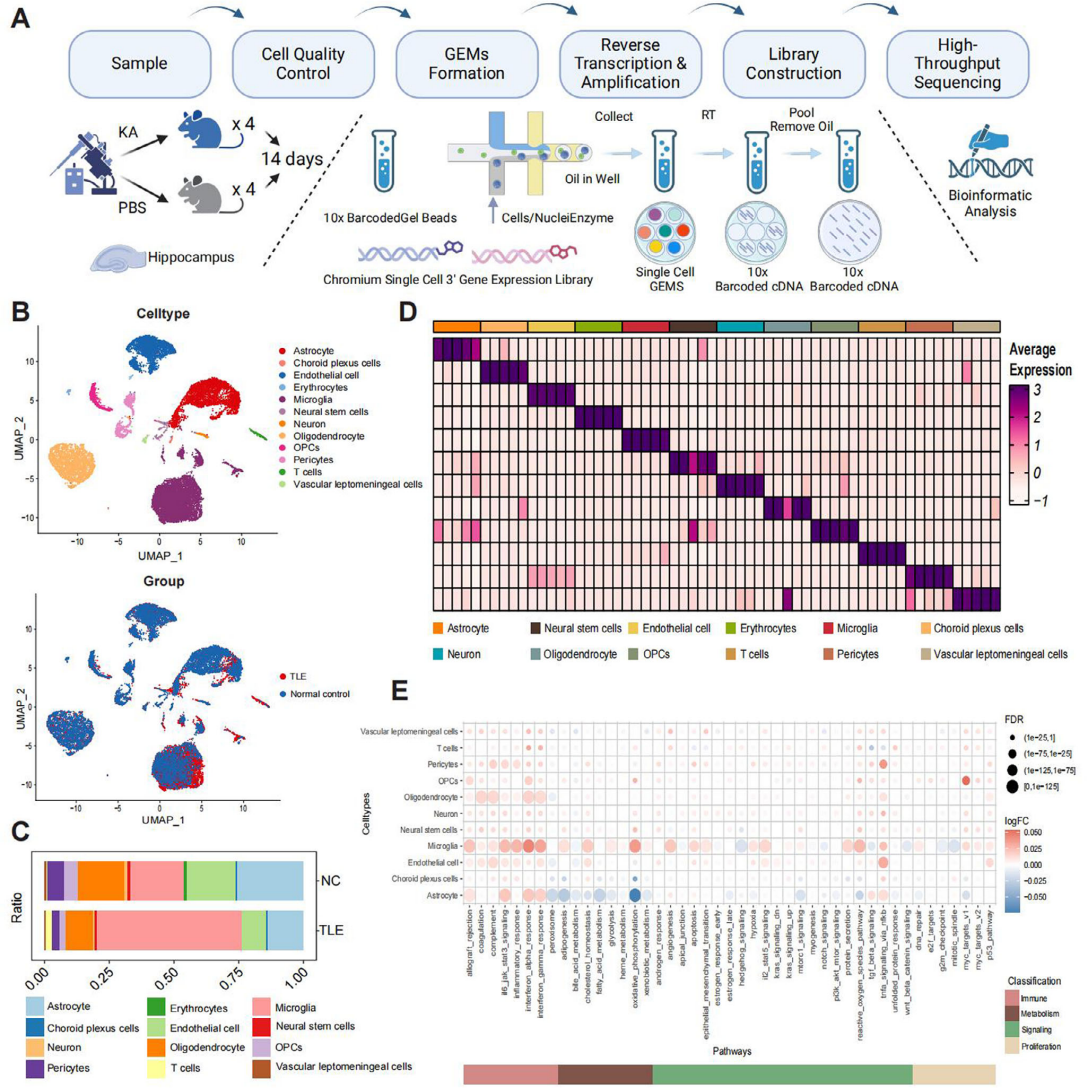
Single‐cell RNA sequencing revealed the characteristics of cellular composition in the hippocampus under temporal lobe epilepsy and marked microglia reactivity. A) Schematic representation of scRNA‐seq analysis of hippocampal samples (injection side) from TLE mice (*n* = 4) and PBS control mice (*n* = 4). Created by BioRender.com. B) UMAP clustering of cell populations. Upper panel: UMAP visualization of all single‐cell RNA‐seq data colored by annotated cell type, revealing 12 major hippocampal cell types. Different colors denote distinct cell types. Lower panel: UMAP visualization of the same cells colored by experimental group, showing cells derived from the TLE group (red) and control group (blue). C) Bar chart showing the proportion of each cell subtype in TLE and control groups, with a marked increase in microglia in the TLE group. D) The heatmap displays the average expression levels of selected marker genes across 12 identified cell types, revealing distinct global gene expression patterns that underscore functional heterogeneity among these cellular populations. The color gradient reflects relative expression levels, with more intense hues indicating higher expression. E) Dot plot illustrating the differentially enriched functional pathways related to immunity, metabolism, signaling, and proliferation in the global cell type between TLE and NC hippocampus.

Single‐cell RNA sequencing (scRNA‐seq) was conducted on hippocampal‐derived cell suspensions from TLE mouse models. The scRNA‐seq data were categorized into two groups: TLE and blank control, with four hippocampal samples in each group from mice treated with kainic acid (KA) and phosphate‐buffered saline (PBS), respectively (**Figure** **2**A). Of the initial cell counts, 13,121 and 11,433 cells in the control and TLE groups, respectively, met the quality control standards, with a base error rate of less than 0.02%. Raw data statistics and quality assessment are provided in Figures S1 and S2 (Supporting Information). Following batch effect removal and clustering analysis based on transcriptome similarity, the cells were divided into 12 major cell types: astrocytes, choroid plexus cells, endothelial cells, erythrocytes, microglia, neural stem cells, neurons, oligodendrocytes, oligodendrocyte precursor cells, pericytes, T cells, and vascular leptomeningeal cells (Figure 2B). Annotation and definition of marker genes for each cell type are provided in Figure S3 (Supporting Information).

The cell population analysis revealed that microglia were the predominant cell type overall. Compared with the control group, the TLE group exhibited substantial microgliosis and neuronal loss (Figure 2C). Since single‐cell analysis was performed on cell suspensions to capture a broad range of cell types, neurons were relatively underrepresented. The heat map revealed distinct global expression patterns across cell types, highlighting marked functional or activity‐based heterogeneity among different cellular populations (Figure 2D). Functional analysis of cellular roles in immunity, metabolism, signaling, and proliferation showed significant functional alterations of microglia. Specifically, microglia exhibited significant enrichment in immune‐ and inflammation‐related pathways, including complement activation, oxidative stress, interferon (IFN) response, and IL6‐JAK‐STAT3 signaling, emphasizing their central role in inflammatory regulation within the epileptic environment. Concurrently, their heightened activity in metabolic pathways, such as glycolysis and oxidative phosphorylation, suggested an abnormal energy demand under pathological conditions (Figure 2E). These findings indicate that microglia play a multifaceted role in disease progression.

### 2.2 Apolipoprotein E Upregulation in Epileptic Conditions: Microglia as the Primary Carrier

**Figure 3:**
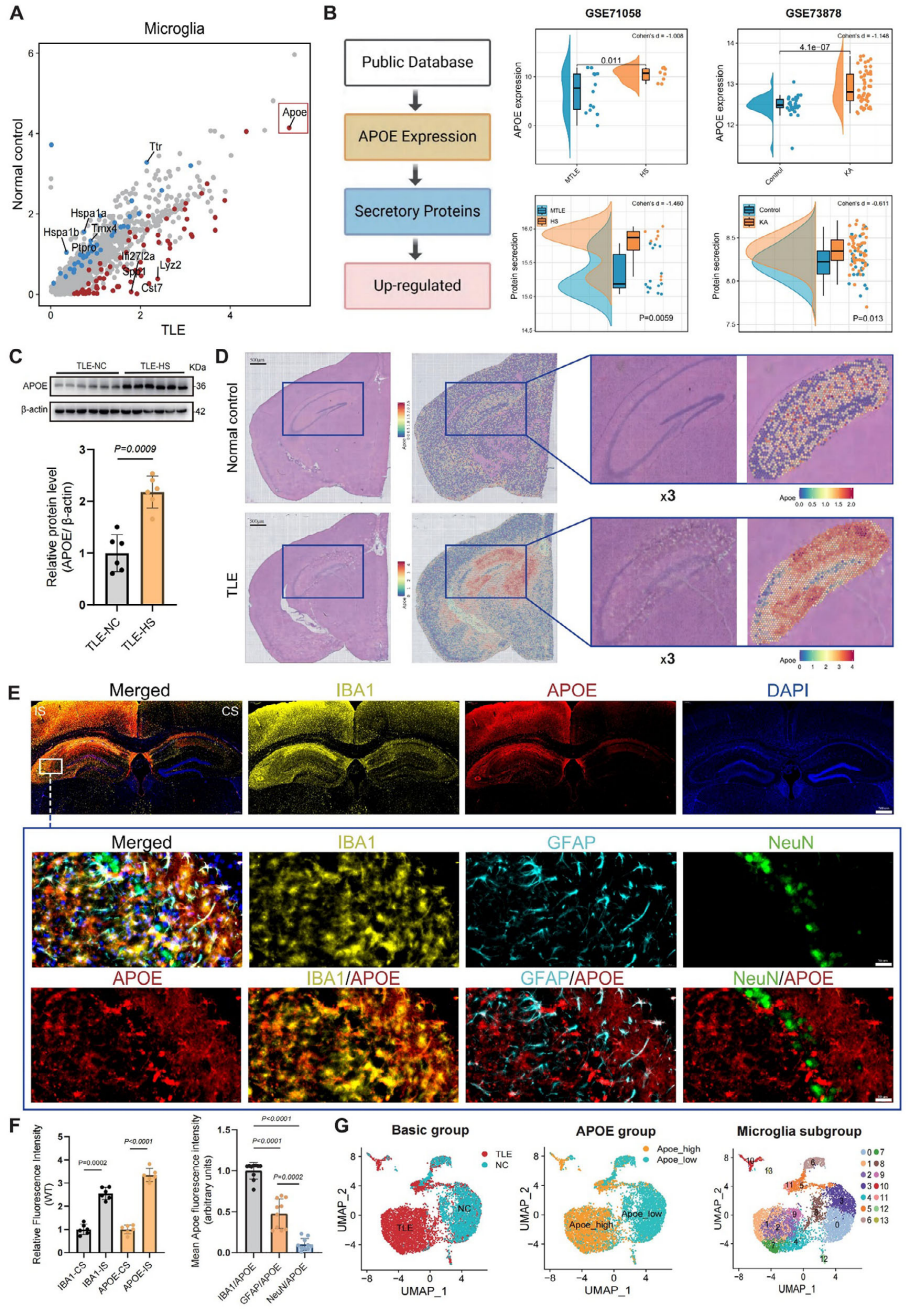
APOE was significantly elevated in the hippocampus, with microglia serving as the principal carrier. A) Scatterplot depicting differentially expressed genes in microglia between TLE and control groups. Axes represent gene expression levels in TLE (x‐axis) vs controls (y‐axis). Each dot denotes an individual gene; red dots indicate significantly upregulated genes (*p* < 0.01, |log2FC| > 1), while blue dots denote significantly downregulated genes (*p* < 0.01, |log2FC| > 1). The five genes exhibiting the highest up‐ and downregulated fold changes are labelled. B) Schematic of the bioinformatic analysis pipeline (left) and violin plots validating *APOE* upregulation at the transcript level in independent datasets (right). Violin plots illustrate increased *APOE* mRNA expression (top) and activation of secretory protein pathway gene signatures (bottom), consistent across both human (GSE71058) and mouse data (GSE73878). The standardized effect size (Cohen's d) is displayed in the top‑right corner. C) APOE expression was markedly elevated in the hippocampus of TLE patients with hippocampal sclerosis (TLE‐HS) relative to those without sclerosis (TLE‐NC). Upper: Results of Western blot analysis display the levels of APOE and loading control (β‐actin) in TLE‐HS hippocampal tissues. Lower: Quantification of the relative expression of APOE/β‐actin according to the Western blot data. D) Spatial transcriptomics of a PBS control mouse and a KA‐induced TLE mouse model reveals region‐wide expression of *APOE* mRNA across the whole brain (left) and hippocampus (right). E) Immunofluorescence staining of brain tissue sections from TLE mice (*n* = 6), IS and CS denote the KA‐injection (ipsilateral) and PBS‐injection (contralateral) sides, respectively. Top: Representative images of APOE protein (red) in coronal brain sections from wild‐type mice 14 days post‐KA injection (scale bar, 500 µm), co‐stained with IBA1 (yellow, microglia) and DAPI (blue, nuclei). Bottom: Higher magnifications images of APOE protein (red) in the hippocampal CA3 lesion (Scale bar,50 µm) showing co‐localization with microglia (IBA1, yellow), astrocytes (GFAP, cyan), and neurons (NeuN, green); nuclei were counterstained with DAPI (blue). F) Quantification of relative APOE immunofluorescence intensity in different cell types based on E. Left: Relative fluorescence intensities of IBA1 and APOE in hippocampal tissues were significantly higher on the IS of TLE mice than on the CS (*n* = 6). Right: The mean fluorescence intensity of APOE was calculated in each cell type (*n* = 10, each dot represented an individual cell). G) UMAP visualization of microglia clusters colored by tissue origin (left), *APOE* expression levels (middle), and cell type (right). The data are shown as the mean ± SD. Statistical significance was determined by paired Student's t‐test (B and F‐left), independent‑samples t‑test (C), and one‐way ANOVA followed by Tukey's HSD test (F‐right).

Differential gene expression of microglial clusters between the TLE and control groups identified the lipid transport gene *APOE* as the most prominent differentially expressed gene (DEG), which was significantly upregulated in the microglia of the TLE group (**Figure** **3**A). Considering that APOE is a secreted protein, we further analyzed accessible public datasets to evaluate *APOE* expression and its activation of secretory protein pathways. The results indicated that dentate granule cells in patients with TLE‐HS showed significantly higher *APOE* expression and enhanced activation of secretory pathways than patients with TLE without HS. A similar phenomenon was observed when comparing the hippocampi of KA‐injected mice to the contralateral hippocampi (Figure 3B). Validation using the collected clinical samples confirmed that APOE protein expression was significantly higher in the hippocampal tissues of patients with TLE‐HS than in those of patients with TLE without HS (Figure 3C). Spatial RNA sequencing further revealed substantially higher *APOE* expression across the ipsilateral hemisphere in the TLE group than in controls, particularly within the hippocampus and thalamus, with specific localization in the CA1 and CA3 regions (the exposed regions of the skull), which are crucial in epilepsy progression (Figure 3D).

To identify the primary cellular contributors to APOE upregulation in TLE, we examined its expression across different cell types. Under normal physiological conditions, APOE is predominantly expressed in astrocytes, as confirmed from the scRNA‐seq data (Table S1, Supporting Information). In epilepsy, both astrocytes and neurons exhibit upregulated *APOE* gene expression; however, their relative contributions decrease when compared with other cell types. Conversely, microglia demonstrate the most significant increase in *APOE* expression, both intrinsically and relative to other cell types (Table S1, Supporting Information). Immunofluorescence staining of coronal brain sections from wild‐type mice subjected to a 14‐day KA‐induced chronic epilepsy (WT+KA) revealed that APOE expression and microglia (ionized calcium‐binding adaptor molecule 1 (IBA1)+) were markedly higher on the ipsilateral (injected) side (IS) than on the contralateral (non‐injected) side (CS). This observation aligns with the transcriptional trends validated in the GSE73878 dataset (Figure 3B,E,F). Additionally, compared with astrocytes (glial fibrillary acid protein (GFAP)+) and neurons (NeuN+), APOE expression was more pronounced in microglia (Figure 3E,F), suggesting that microglia are the primary contributors of APOE upregulation observed in patients with TLE. Consequently, we conducted a subgroup analysis to further investigate the role and function of APOE expression in microglia (Figure 3G).

### 2.3 Apolipoprotein E Gene Regulates the Differentiation and Function of Microglia in Temporal Lobe Epilepsy

**Figure 4:**
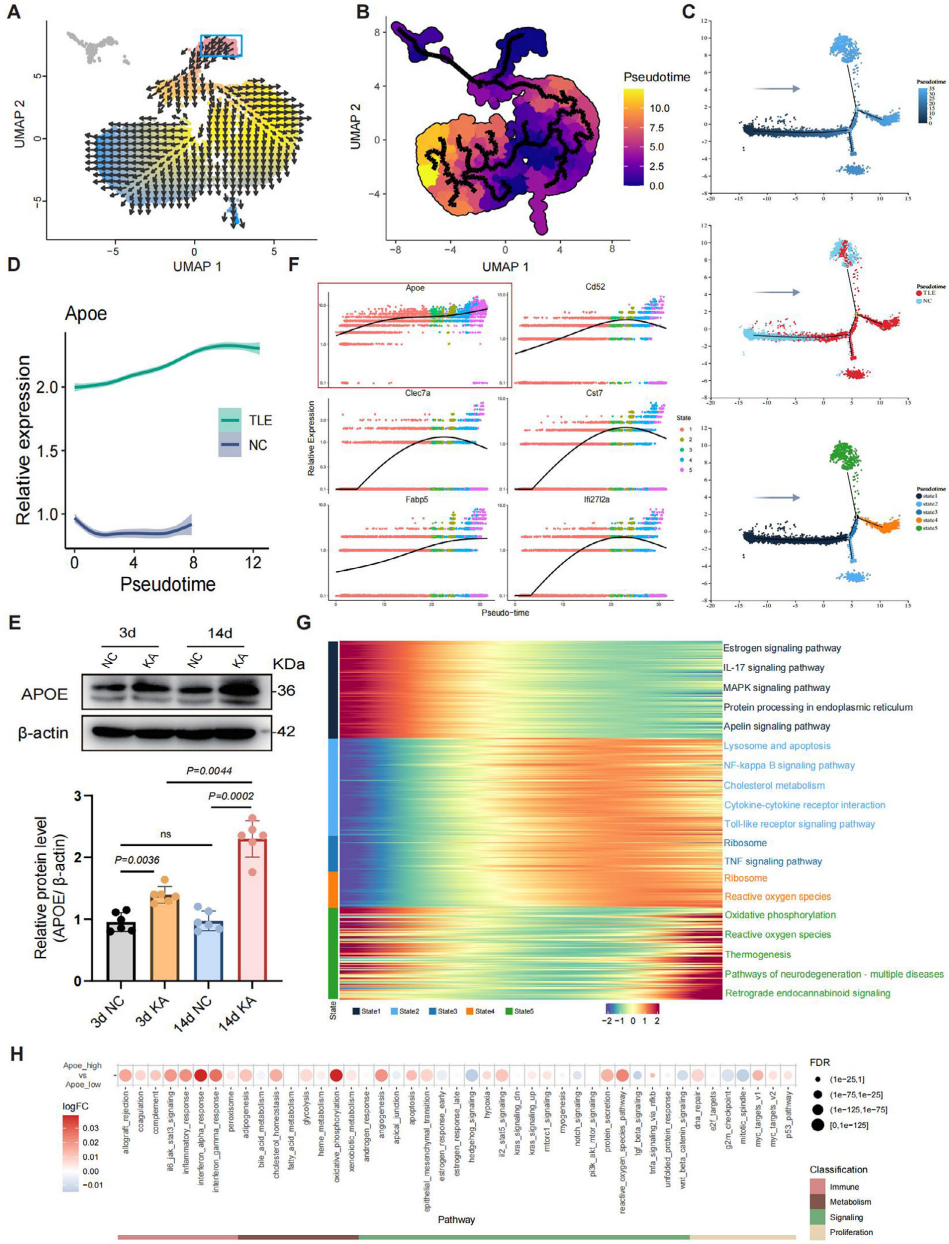
APOE expression and microglial differentiation. A) A vector representation of microglial developmental trajectories generated with VECTOR. Cell pseudotime is depicted using a color gradient from red (early) to blue (late). B) Pseudotime analysis of microglia. C) Three panels displaying pseudotime‐based cellular developmental trajectories: pseudotime (top), grouping (middle), and state (bottom). Each dot represents a single cell arranged along a continuum according to its pseudotime value, with numbered black circles denoting differentiation nodes. This trajectory illustrates the progression of cells from an early to a late developmental state. D) Relative expression of *APOE* in microglia over pseudotime for the TLE group (green) and control group (blue). E) Expression of APOE in the ipsilateral hippocampus of TLE and PBS control mice at 3 and 14 days (*n* = 6 per group per time point). Upper: Results of Western blot analysis display the levels of APOE and loading control (β‐actin) in TLE‐HS hippocampal tissues. Lower: Quantification of the relative expression of APOE/β‐actin according to the Western blot data. The data are shown as the mean ± SD. Statistical significance was determined by two‐way ANOVA followed by Tukey's HSD test. F) Overall pseudotime‐dependent differential gene expression scatter plots. Each point is color‐coded by differentiation state, with the horizontal axis representing temporal progression from early to late. The plot highlights the expression trajectories of the top six genes; genes that exhibit an earlier increase/decrease in expression are activated/suppressed sooner. G) Gene expression patterns and related pathways across pseudo‐time in the five states of microglia. Different colors indicate distinct microglia states. H) Dot plot illustrating the differentially enriched functional pathways related to immunity, metabolism, signaling, and proliferation between microglia with high *APOE* expression and with low *APOE* expression.

VECTOR analysis of microglial lineage differentiation identified cluster 6 as the progenitor population. From this origin, microglia differentiated along two primary trajectories, one leading to normal microglial subpopulations and the other to TLE‐associated subsets (Figures 3G and 4A). Subsequent pseudotime analysis using Monocle3 with cluster 6 as the developmental starting point revealed that TLE‐associated microglia predominantly reside at the terminal differentiation stages (Figures 3G and 4B). Visualization of the microglial pseudotemporal differentiation trajectories, categorized by pseudotime, group, and state, revealed a distinct separation of activated microglia along the predicted pseudotime progression (**Figure** **4**C). Microglia from the TLE group predominantly occupied the mid‐to‐late pseudotime stages, indicating disease‐specific differentiation. Control microglia were primarily situated in the early pseudotime stages; however, their presence in terminal stages suggests a dynamic balance between ‘protective’ and ‘pro‐inflammatory’ states during later disease phases, such as chronic inflammation or neurodegeneration.

Additionally, *APOE* gene expression levels exhibited dynamic changes along the pseudotime trajectory. In the TLE group, *APOE* expression was not only significantly elevated but also continued to increase throughout pseudotime, reflecting sustained microglia reactivity under pathological conditions (Figure 4D). Analysis of APOE protein expression in the ipsilateral hippocampus of TLE mice at 3 days (common latent period) and 14 days (chronic phase) post‐induction^[^ ^17^ ^]^ confirmed that APOE expression increased with disease progression, corroborating the pseudotime analysis findings (Figure 4E).

Differential gene expression trends at the overall differentiation branch points based on the five previously defined states (Figure 4C) identified the top six key genes (Figure 4F). Unlike the other genes, *APOE* expression consistently exhibited an upward (activation) trend over time without any decline (suppression), suggesting that *APOE* plays a pivotal role in microglia reactivity and differentiation in TLE. Further analysis of gene expression patterns and related pathways revealed distinct functional profiles across different microglial states (Figure 4G). Cells in state 1 were primarily involved in hormone regulation, protein synthesis, energy metabolism, and vascular homeostasis, with no obvious signs of activation or reactivity. States 2–4 shared similar gene expression signatures but exhibited unique functional characteristics. Specifically, microglia in state 2 transitioned into inflammatory activation, immune response, and coordinated lipid metabolism; those in state 3 exhibited active ribosomal pathways, suggesting a proliferative or secretory status and implying that state 3 is a proliferative‐inflammatory transitional state; and finally, those in state 4 were associated with cellular damage response and oxidative stress. Furthermore, microglia in state 5 represented a late‐stage response dominated by metabolic reprogramming and neurodegeneration‐related pathways, suggesting their involvement in chronic neuropathological changes and neurorepair mechanisms. Notably, activation of the endocannabinoid signaling pathway, which regulates synaptic excitability and inflammation in epilepsy, was prominent in this state. Preliminary functional analysis of microglia with high *APOE* expression revealed significant enrichment in immune‐related pathways, mirroring the characteristic features of TLE‐associated microglia (Figure 4E,H). Given this strong immunological signature, we subsequently investigated the possible inflammatory mechanisms mediated by *APOE*‐expressing microglia.

### 2.4 Overexpression of Apolipoprotein E Enhances Microglial Inflammatory Responses

**Figure 5:**
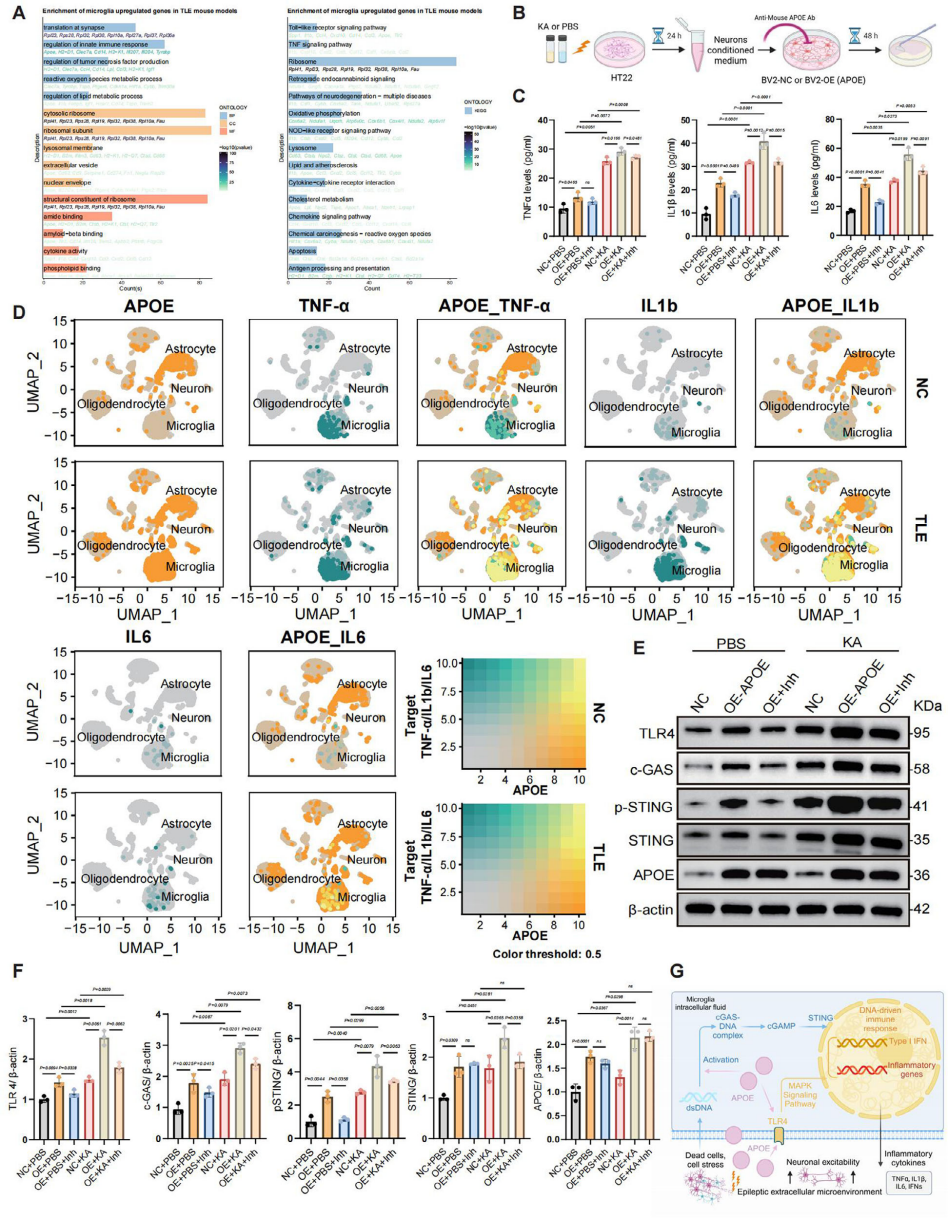
APOE promotes inflammatory reactivity in microglia. A) GO and KEGG enrichment analyses of differentially expressed genes between *APOE*‐overexpressing microglia and microglia with low *APOE* expression. B) Schematic illustration of the co‐culture workflow: HT22 neurons were treated with either PBS or KA for 24 h; the resulting HT22‐conditioned medium was then applied to normal or *APOE*‐overexpressing BV2 microglia for 48 h. In addition, two inhibitor groups were established by adding the Anti‑Mouse APOE Monoclonal Antibody together with HT22‑conditioned medium to the *APOE*‑overexpressing microglia for 48 h as well. Conditioned medium was collected for ELISA, and cells were used to validate pathway protein expression. C) Levels of the cytokines TNFα, IL‐1β, and IL‐6 in the conditioned medium. D) UMAP visualization showing the expression of *APOE* and inflammatory cytokine genes (*TNFα*, *IL‐1β*, and *IL‐6*) across various cell types, and the degree of overlap between *APOE* and these inflammatory genes. E) Western blot analysis displaying protein expression of TLR4, cGAS, phosphorylated‐STING (p‐STING), STING, and APOE in microglia isolated from PBS‐ or KA‐treated groups, with β‐actin as a loading control. F) Quantification of the relative expression of TLR4, cGAS, p‐STING, STING, and APOE normalized to β‐actin based on the Western blot data. G) A proposed model illustrating the potential mechanism by which APOE enhances inflammatory activation in microglia. Created by BioRender.com. All experiments were independently repeated three times. The data are shown as the mean ± SD. Statistical significance was determined by two‐way ANOVA followed by Tukey's HSD test (C and F).

Gene Ontology (GO) and Kyoto Encyclopedia of Genes and Genomes (KEGG) enrichment analyses of *APOE*‐overexpressing microglia revealed significant enrichment in immune‐ and inflammation‐related pathways, including the innate immune response, cytokine activity, tumor necrosis factor (TNF) signaling, and Toll‐like receptor (TLR) signaling pathways (**Figure** **5**A). Subsequently, we quantified the levels of pro‐inflammatory cytokines (interleukin (IL)‐6, IL‐1β, and TNF‐α) secreted by microglia in different co‐culture models to validate their functional phenotype (Figure 5B). Treatment of either control (BV2) or *APOE*‐overexpressing microglial cells (BV2‐OE) with conditioned media from neurons (HT22) exposed to KA for 24 h resulted in significantly elevated levels of TNFα, IL‐1β, and IL‐6 after 48 h, compared with the PBS‐treated control group (Figure 5C). Notably, *APOE*‐overexpressing microglia treated solely with conditioned media from PBS‐treated neurons also exhibited significantly increased TNFα, IL‐1β, and IL‐6 levels (Figure 5C). However, application of the APOE inhibitor elicited a pronounced reduction in their expression levels. We further verified *TNFα*, *IL‐1β*, and *IL‐6* gene expression in microglia using scRNA‐seq, along with their co‐expression with *APOE* (Figure 5D). Enzyme‐linked immunosorbent assay (ELISA) analysis of KA‑ vs PBS‑treated HT22 neurons revealed elevations in TNF‑α, IL‑1β, and IL‑6, although their absolute concentrations remained low (Figure S4, Supporting Information). We ruled out any potential interference from neuronal (HT22) cytokine secretion observed in the ELISA experiments through this validation. Under TLE conditions, *TNFα*, *IL‐1β*, and *IL‐6* were predominantly expressed in microglia (rather than in neurons or astrocytes) and co‐localized with *APOE* (Figure 5D). Collectively, these findings confirmed that upregulated *APOE* expression in microglia strongly promotes inflammatory cytokine secretion.

Building on previous findings, we assessed several keys signaling pathways and protein levels in microglia under different co‐culture conditions (Figure 5E,F). Compared with the control group (BV2‐NC+PBS), the protein expression levels of TLR4 and the c‐GAS/STING pathway significantly and progressively increased in the *APOE*‐overexpressing (BV2‐OE+PBS), the KA‐stimulated (BV2‐NC+KA), and most notably, the combined *APOE* overexpression and KA‐stimulation groups (BV2‐OE+KA), suggesting a clear synergistic effect. After the addition of the APOE inhibitor, all related pathways were suppressed (Figure 5E,F). Based on these observations, we proposed a schematic illustration of the potential mechanisms underlying microglial inflammatory reactivity in TLE (Figure 5G).

### 2.5 Interactions Between *APOE*‐Overexpressing Microglia and Other Cell Types

**Figure 6:**
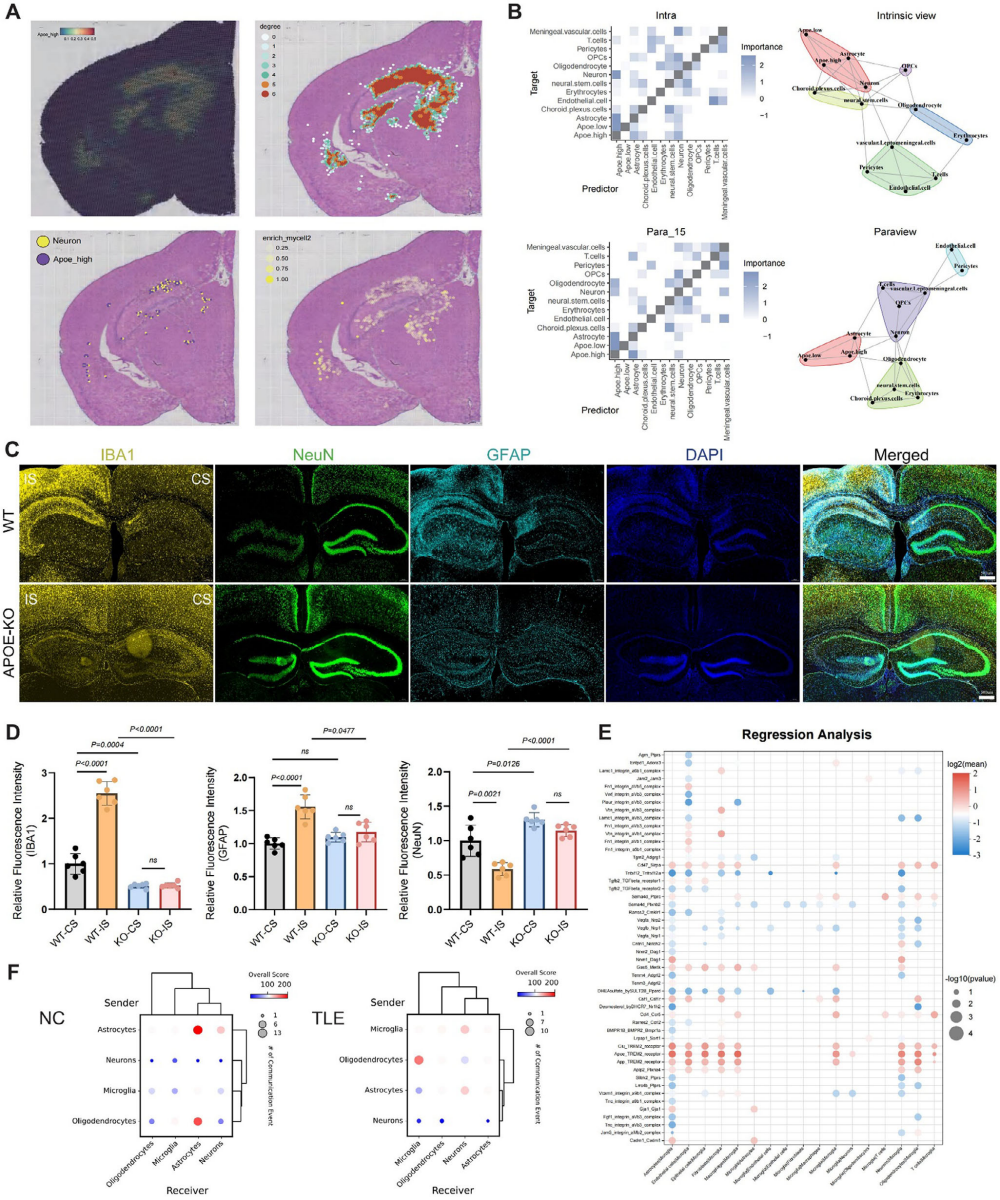
APOE affects intercellular interactions. A) Comparative cell network analysis: from top left to bottom right the panels respectively display: (i) the spatial distribution and expression patterns of microglia with high *APOE* expression in TLE brain sections; (ii) homotypic cell–cell interactions among high APOE‐expressing microglia in TLE; (iii) heterotypic cell–cell interactions between high *APOE*‐expressing microglia and neurons in TLE; and (iv) the enrichment of high *APOE*‐expressing microglia with surrounding neurons, where darker colors indicate a higher abundance of target cell pairs. B) Spatial co‐localization analysis revealing the cellular neighborhood composition and interactions among different cell types in TLE brain tissue. The top panels display a heatmap of interaction intensities among cell pairs within the same spatial unit and a network community visualization depicting these cellular interaction patterns. The bottom panels show a heatmap and a corresponding network community visualization of interaction intensities among cell pairs within a 15‐unit neighbouring spatial range. C) Immunofluorescence staining of brain tissue sections from TLE mice, IS and CS denote the KA‐injection (ipsilateral) and PBS‐injection (contralateral) sides, respectively. Top: Representative images of microglia (IBA1, yellow), neurons (NeuN, green), and astrocytes (GFAP, cyan) in coronal brain sections from wild‐type mice 14 days post‐KA injection (scale bar, 500 µm). Bottom: Representative images of microglia (IBA1, yellow), neurons (NeuN, green), and astrocytes (GFAP, cyan) in coronal brain sections from *APOE* ^−/−^ mice 14 days post‐KA injection (scale bar, 500 µm); nuclei were counterstained with DAPI (blue) (*n* = 6 each group). D) Quantification of relative microglia (IBA1), neurons (NeuN), and astrocytes (GFAP) immunofluorescence intensity in different groups based on C. Three markers (IBA1, GFAP, and NeuN) were analysed by two‑way ANOVA with side (IS vs CS) and genotype (WT+KA vs *APOE* ^−/−^+KA) as factors, followed by Tukey's HSD test. The data are shown as the Mean ± SD. E) Bubble plot depicting the average expression levels and p‐values of ligand–receptor pairs. The x‐axis represents cell type interactions, and the y‐axis denotes the ligand–receptor pairs. F) Bubble plots of cellular metabolic communication based on single‐cell transcriptomics for the NC group (left panel) and TLE group (right panel). The x‐axis indicates sender cell types, while the y‐axis represents receiver cell types.

Spatial transcriptomic sequencing revealed a high concentration of *APOE*‐expressing microglia in the hippocampus (CA1 and CA3 regions) of the TLE mouse model (**Figure** **6**A). Both homotypic and heterotypic cellular network analyses revealed significantly high clustering of *APOE*‐expressing microglia in the hippocampal CA1 region (the area of most severe sclerosis) and close colocalization with neurons (Figure 6A). Multi‐view intercellular spatial modeling (MISTy) analysis, a spatial transcriptomic method used to uncover communication and interaction patterns among different cell types (Figure 6B), further revealed strong connections between *APOE‐*expressing microglia and neurons/astrocytes through both intracellular gene expression (intra) and paracrine signaling (para_15), suggesting a prominent communication network (Figure 6B). These findings indicate that *APOE* overexpression in microglia may significantly influence the behavior of the surrounding neuronal and astrocytic populations in TLE. To further explore this, we established a 14‐day TLE model using *APOE* knockout mice (*APOE* ^−/−^+KA) and performed immunofluorescence staining on hippocampal coronal sections, comparing them with WT+KA mice (Figure 6C,D). In WT+KA mice, the IS hippocampus showed significantly increased microglia reactivity, neuronal loss, and astrogliosis compared with the CS; however, *APOE* ^−/−^+KA mice exhibited neuronal damage without significant differences in microglial or astrocytic reactivity compared with the CS hippocampus. Between‐group comparisons demonstrated significantly reduced microglia reactivity and neuronal loss in both the IS and CS hippocampi of *APOE* ^−/−^+KA mice compared with those of WT+KA mice, alongside less pronounced astrogliosis in the IS. These findings suggest that *APOE* knockdown may attenuate neuroinflammation and neuronal injury in the hippocampus following KA‐induced epilepsy.

Additionally, we conducted regression analyses to elucidate microglial interactions with other cell types and to identify potential ligand‐receptor pairs in TLE (Figure 6E). Single‐cell transcriptomic data and metabolite databases were used for MEBOCOST metabolic communication analysis, which predicted metabolic interactions among various cell populations (Figure 6F). Under normal physiological conditions, astrocytes predominantly regulate brain tissue metabolism. However, during TLE, communications between microglia and neurons are notably activated, suggesting a metabolic shift. These findings highlight a strong spatial and functional association between *APOE*‐overexpressing microglia and neurons in epileptic hippocampal regions, suggesting a potential regulation of neuronal excitability. Moreover, the observed microglial shift in TLE underscores the need for further investigation into the role of *APOE* in metabolic reprogramming under epileptic conditions.

### 2.6 *APOE* Overexpression in Microglia Enhances Neuronal Excitability and Exacerbates Epileptic Pathology

**Figure 7:**
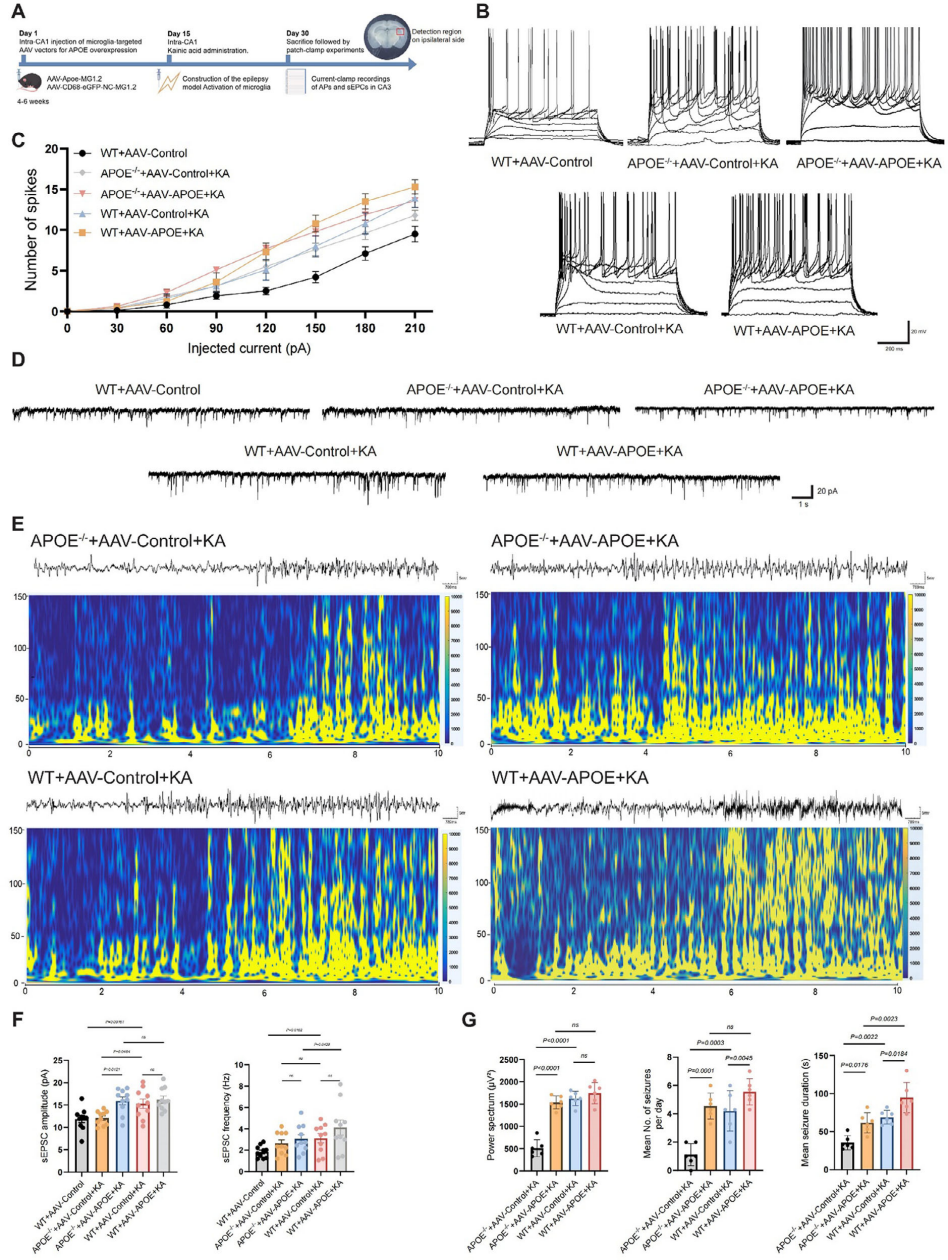
Electrophysiological pathology in TLE associated with APOE‐overexpressing microglia. A) Schematic diagram illustrating the patch‐clamp current‐clamp recording of action potentials (APs) and spontaneous excitatory postsynaptic currents (sEPSCs) in CA3 pyramidal neurons. B, C): AP analysis; B) displays representative current‐clamp traces of APs recorded from neurons in five conditions (WT+AAV‐Control, *APOE* ^−/−^+AAV‐Control+KA, *APOE* ^−/−^+AAV‐*APOE*+KA, WT+AAV‐Control+KA, and WT+AAV‐*APOE*+KA). Scale bars: 20 mV (vertical) and 200 ms (horizontal); C) shows the number of spikes generated at increasing current steps for each group, illustrating differences in neuronal excitability. (*n* = 10 valid cells recorded per group). D, F): sEPSCs analysis; D) displays representative sEPSCs recordings from five groups (WT+AAV‐Control, *APOE* ^−/−^+AAV‐Control+KA, *APOE* ^−/−^+AAV‐*APOE*+KA, WT+AAV‐Control+KA, and WT+AAV‐*APOE*+KA). The scale bars represent 20 pA (vertical) and 1 s (horizontal). F) shows the corresponding quantifications of sEPSCs amplitude (left) and frequency (right). E, G) display intracranial EEG recordings from 14‐day TLE mice of different types (*APOE* ^−/−^+AAV‐Control+KA, *APOE* ^−/−^+AAV‐*APOE*+KA, WT+AAV‐Control+KA, WT+AAV‐*APOE*+KA) (*n* = 6 per group); E) shows representative epileptic EEG traces and their corresponding time‐frequency plots for the five groups. G) presents the quantification of power spectral energy in the high‐frequency (80‐150 Hz) oscillation range (left), seizure frequency (middle) and duration (right) recorded continuously over 24 h for three consecutive days in different groups of TLE mice. The data are shown as the mean ± SD. Statistical significance was determined by two‐way repeated‐measures (C) and two‐way ANOVA (F and G), followed by Tukey's HSD test.

To examine the effect of *APOE*‐overexpressing microglia on neuronal excitability at the cellular level, we targeted microglia in the hippocampal region of WT mice using adeno‐associated virus (AAV)‐mediated APOE overexpression, followed by the induction of a 14‐day chronic TLE model (WT+AAV‐*APOE*+KA). Four additional groups were included: WT mice receiving AAV‑Control and PBS (WT+AAV‑Control), *APOE* ^−/−^ mice receiving AAV‑Control and KA (*APOE* ^−/−^+AAV‐Control+KA), *APOE* ^−/−^ mice receiving AAV‑*APOE* and KA (*APOE* ^−/−^+AAV‐*APOE*+KA), and WT mice receiving AAV‑Control and KA (WT+AAV‐Control+KA) (**Figure** **7**A). Compared with the WT+AAV‐Control group, the *APOE* ^−/−^+AAV‐*APOE*+KA, WT+AAV‐Control+KA, and WT+AAV‐*APOE*+KA groups exhibited a significant increase in the number and frequency of action potentials (APs) in CA3 pyramidal neurons (Figure 7B,C). Furthermore, the WT+AAV‐*APOE*+KA group showed a trend toward enhanced neuronal excitability relative to the WT+AAV‐Control+KA group. However, the *APOE* ^−/−^+AAV‑Control+KA group exhibited reduced neuronal excitability compared with the *APOE* ^−/−^+AAV‑*APOE*+KA and WT+AAV‑Control+KA groups. Neuronal firing rates varied markedly among treatment groups and across current intensities, reflecting significant main effects of both factors and their interaction. Across all current injections, both *APOE* ^−/−^+AAV‑*APOE*+KA and WT+AAV‐*APOE*+KA consistently exhibited higher spike counts than the other three groups. Additionally, compared with the WT+AAV‐Control group, both the WT+AAV‐Control+KA and WT+AAV‐*APOE*+KA group showed a significant increase in the frequency and amplitude of spontaneous excitatory postsynaptic currents (sEPSCs) in CA3 pyramidal neurons (Figure 7D,F). The WT+AAV‐*APOE*+KA group exhibited a significantly higher frequency of sEPSCs than the AAV‐Control +KA group. Conversely, both the frequency and amplitude of sEPSCs were significantly reduced in the *APOE* ^−/−^+AAV‐Control+KA group compared with the WT+AAV‐Control+KA group. In the *APOE* ^−/−^+AAV‐*APOE*+KA group, sEPSCs amplitude and frequency were comparable to those observed in WT+AAV‐*APOE*+KA. These findings indicate that APOE‐overexpressing microglia increase neuronal excitability at the cellular level in the TLE model, while *APOE* deletion partially attenuates this neuronal hyperexcitability.

Subsequently, we investigated whether *APOE* and *APOE*‐overexpressing microglia enhance neuronal firing and exacerbate chronic epilepsy pathology in animal models. For this purpose, we established four 14‐day TLE mouse models: *APOE* ^−/−^+AAV‑Control+KA, *APOE* ^−/−^+AAV‐*APOE*+KA, WT+AAV‑Control+KA, and WT+AAV‐*APOE*+KA. The in vivo experiments followed the same procedural workflow as the in vitro studies described above (Figure 7A). All four groups included in the analysis successfully developed status epilepticus (SE) following stereotactic KA injection. The severity of SE, assessed by the modified Racine scale over the subsequent two‐hour monitoring period, is presented in Figure S5 (Supporting Information). Using in vivo intracranial electroencephalogram (EEG) monitoring combined with video recordings, we documented seizure‐related EEG signals, average daily seizure frequency, and mean seizure duration (Figure 7E,G). Results showed that the WT+*APOE* ^−/−^+KA group exhibited fewer abnormal discharges with weaker high‐frequency components (80‐150Hz) in the time‐frequency spectrogram (Figure 7E,G; Figure S6, Supporting Information). During seizures, comparison of power spectral energy in the 80–150 Hz high‑frequency oscillation band across the four groups showed that the WT+AAV‑*APOE* + KA group exhibited the highest spectral energy, whereas the *APOE* ^−/−^ + AAV‑Control + KA group displayed the lowest high‑frequency oscillation energy (Figure 7G; Figure S6, Supporting Information). The *APOE* ^−/−^+AAV‐*APOE*+KA and WT+AAV‐Control+KA groups exhibited significantly greater spectral power in the high‐frequency (80–150 Hz) band than the *APOE* ^−/−^+AAV‐Control+KA group, indicating that both microglia‐specific and global APOE overexpression contribute to this high‐frequency enhancement. Given that microglia constitute the main source of APOE overexpression, this finding highlights their putative contribution to the electrophysiological changes observed. Furthermore, daily seizure frequency and seizure duration were significantly higher in the three remaining groups than in the *APOE* ^−/−^+AAV‐Control+KA group (Figure 7G). Figure S7 (Supporting Information) presents continuous, three‑day monitoring of seizure severity across the four experimental groups. The WT+AAV‐*APOE*+KA group showed a slightly higher frequency of abnormal discharges and a greater presence of high‐frequency components, along with the highest daily seizure frequency, than the WT+AAV‑Control+KA group (Figure 7E,G). Overall, these findings suggest that *APOE* deletion attenuates the severity of KA‐induced epilepsy, whereas APOE overexpression in microglia exacerbates TLE progression and prolongs seizure duration.

### 2.7 *APOE* Primarily Drives Alterations in Glycerophospholipid Metabolism and Associated Metabolic Derivatives During Temporal Lobe Epilepsy

**Figure 8:**
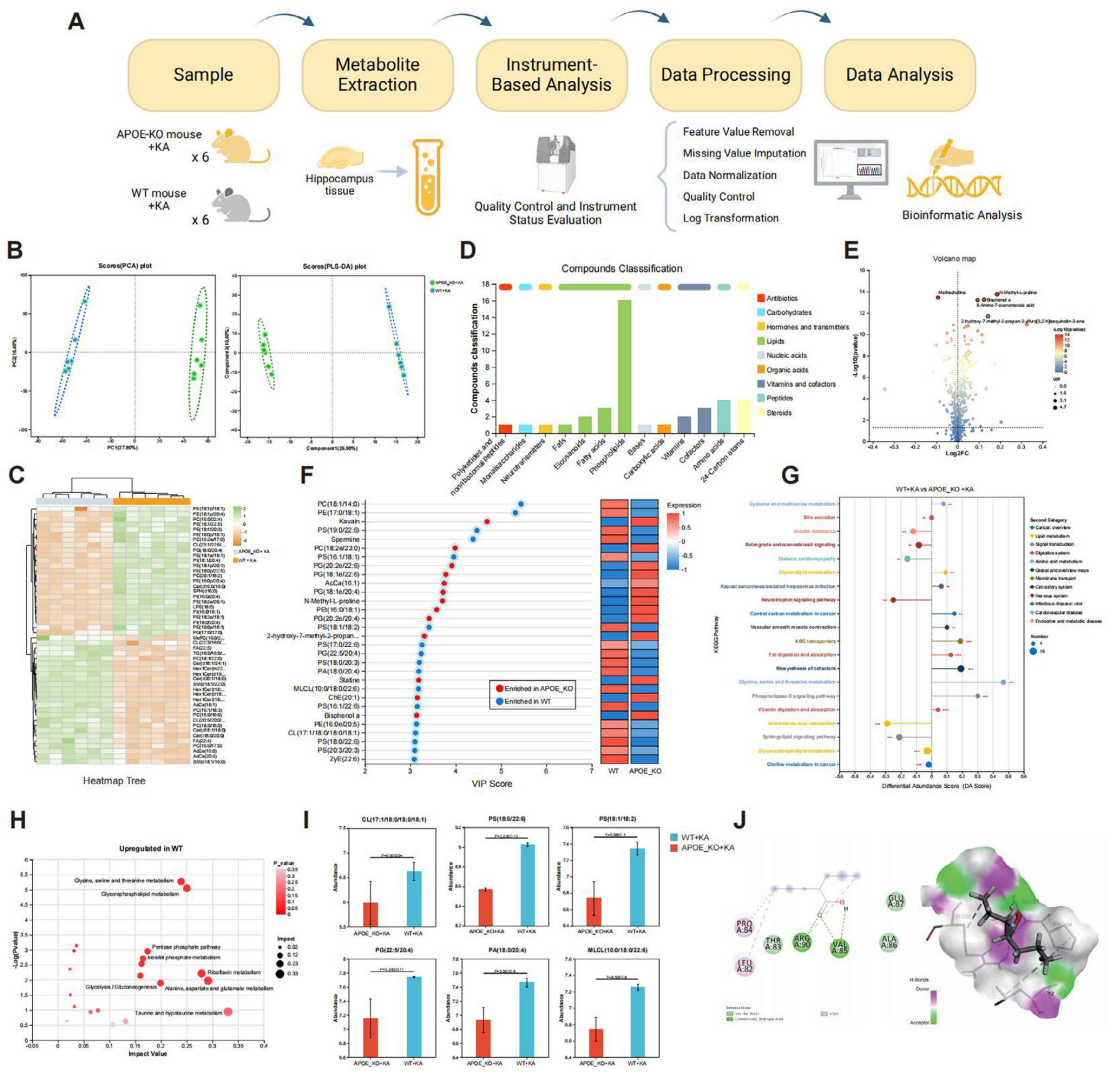
APOE modulates hippocampal metabolism in TLE. A) Schematic representation of the metabolomics and lipidomics study on hippocampal samples (injection side) from different TLE mouse models (*APOE* ^−/−^ + KA and WT + KA) (*n* = 4 per group). Created with BioRender.com. B) PCA score plot illustrating the clustering and dispersion of samples from the *APOE* ^−/−^ + KA and WT + KA groups. C) Heatmap of differential metabolites between *APOE* ^−/−^ + KA and WT + KA groups, showing the trends in metabolite changes. D) Bar chart categorizing the differential metabolites between the *APOE* ^−/−^ + KA and WT + KA groups. E) Volcano plot comparing the differential metabolites between *APOE* ^−/−^ + KA and WT + KA groups. F) VIP plot based on the projection of variables along the first principal component. PLS‐DA was used as the supervised model and validated by 7‐fold cross‐validation to assess predictive differences. G) KEGG pathway differential abundance score plot for the differential metabolites between the *APOE* ^−/−^ + KA and WT + KA groups. H) KEGG topology bubble plot displaying significantly upregulated pathways in the WT + KA group compared to the *APOE* ^−/−^ + KA group. I) Bar chart showing the differential expression of six identified key metabolites. J) 3D and 2D visualization of the protein–ligand complex between the selected drug (sodium valproate) and APOE, generated using PyMOL and Ligplot. The left panel displays the 2D docking diagram of the protein and small molecule, and the right panel illustrates the local interaction map. Statistical significance was determined by a two‐sided unpaired Student's t‐test (two‐tailed test).

We performed untargeted metabolomic and lipidomic analyses using two models, *APOE* ^−/−^+KA and WT+KA, to examine the impact of *APOE* expression on hippocampal metabolism during TLE (Figure **8**A). Quality control assessments of the raw metabolomics and lipidomics datasets are presented in Figure S8 (Supporting Information). Principal component analysis (PCA) and partial least‐squares discriminant analysis (PLS‐DA) plots revealed clear intergroup differences with minimal intragroup variability following dimensionality reduction, highlighting *APOE*’s significant impact on metabolic profiles (Figure 8B). The two groups exhibited markedly distinct metabolomic patterns in their differentially expressed metabolites (Figure 8C). In the hippocampal tissue, the metabolites were predominantly distributed among lipids, peptides, and steroids, with lipids being the most abundant (Figure 8D). Specifically, 213 significantly different metabolites (false discovery rate (FDR) < 0.05) were identified: 84, including spermine, methacholine, and isopentyl beta‐*D*‐glucoside, were upregulated in the WT+KA group, and 121, including kavain, *N*‐methyl‐*L*‐proline, and eicosapentaenoic acid, were upregulated in the *APOE* ^−/−^+KA group (Figure 8E). These metabolites are usually involved in functions such as arachidonic acid metabolism and steroid hormone biosynthesis, with notable disruptions in lipid metabolism, including alterations in eicosapentaenoic acid and prostaglandin compounds. This suggests that *APOE* overexpression exacerbates metabolic abnormalities by disrupting lipid homeostasis and modulating inflammation‐related pathways. The Variable Importance in Projection (VIP) scores derived from the PLS‐DA model were utilized to rank the metabolites contributing to differences between the *APOE* ^−/−^+KA and WT+KA groups (Figure 8F). A notable enrichment of glycerophospholipid metabolites, including phosphatidylcholine (PC, 18:1/14:0), phosphatidylethanolamine (PE, 17:0/18:1), and phosphatidylserine (PS, 19:0/22:6), was observed in the WT+KA group.

Subsequent KEGG pathway enrichment analysis of the overall differential metabolites (Figure 8G) and topological critical pathway analysis in the WT+KA group (Figure 8H) were performed to investigate the potential *APOE*‐mediated metabolic pathways in TLE pathogenesis. Compared with the *APOE* ^−/−^+KA group, significant upregulation was identified in metabolic pathways such as glycine, serine, and threonine metabolism; glycerophospholipid metabolism; and the pentose phosphate pathway within the WT+KA epilepsy model. Additionally, activation of alanine, aspartate, and glutamate metabolism further highlights *APOE*’s role in metabolic reprogramming and regulation of neurotransmitter balance during TLE. Enhanced reliance on lipid and amino acid metabolism in epileptic mice underscores this effect. Integrating the VIP scores of key metabolites with the results from KEGG enrichment analysis, six key metabolites significantly regulated by *APOE* and closely associated with epilepsy progression were identified: cardiolipin (CL, 17:1/18:0/18:0/18:1), PS (18:0/22:6, 18:1/18:2), phosphatidylglycerol (PG, 22:5/20:4), phosphatidic acid (PA, 18:0/20:4), and monolysocardiolipin (MLCL(10:0/18:0/22:6)) (Figure 8I). These metabolites are involved in mitochondrial function, neuroinflammation, and membrane excitability changes.

Lastly, we explored potential drug candidates targeting APOE and found that the clinically common antiseizure medication, valproic acid, exhibits a favorable binding affinity for APOE. Molecular docking analysis revealed that the binding energy between APOE and valproic acid is ‐3.6 Kcal mol^−1^. APOE residues VAL85 and ARG90 formed conventional hydrogen bonds with valproic acid, whereas LEU82, PRO84, and ARG90 engaged in alkyl interactions (Figure 8J). These findings suggest that targeting APOE may have therapeutic potential in epilepsy treatment.

## 3 Discussion

Epilepsy is a severe CNS disorder characterized by an imbalance between neuronal excitation and inhibition, leading to heightened neuronal excitability, particularly within the hippocampus.^[^ ^18^ ^]^ Its underlying pathogenesis primarily encompasses the following aspects:

- Imbalance in neuronal excitability: During epileptic seizures, neurons in certain regions of the brain become excessively excited and discharge synchronously, thereby disrupting the normal balance of electrical activity. This may result from abnormalities in ion channel function, neurotransmitter imbalances, or dysregulation of neurotransmitter receptors.^[^ ^19^, ^20^ ^]^
- Inflammatory response involvement: Studies have confirmed that neuroinflammation is a critical mechanism in epilepsy development. Inflammatory mediators such as IL‐1β and TNF‐α can increase neuronal excitability and compromise the function of inhibitory neurons.^[^ ^21^ ^]^
- Neuroplastic changes: Repeated long‐term epileptic seizures induce structural and functional modifications in neurons, further aggravating the imbalance in neuronal excitability and creating a vicious cycle.^[^ ^22^ ^]^

In our study, we observed a significant upregulation of hippocampal APOE expression in both patients and animal models with TLE, with microglia identified as the principal carriers. Elevated APOE in TLE may be associated with microglia reactivity, which, when overexpressing APOE, contributes to neuroinflammation, increased neuronal excitability, and metabolic alterations (particularly in lipid metabolism) that drive TLE progression.

Microglia, as the principal immune sentinel cells in the CNS, have garnered considerable attention because of their functional polarization states in epilepsy pathology.^[^ ^21^, ^23^ ^]^ Notably, these cells become strongly activated during the chronic phase of epilepsy and can selectively prune inhibitory hippocampal GABAergic synapses. This synaptic pruning alters local neural circuits by disrupting the balance between excitatory and inhibitory synapses, ultimately contributing to epilepsy development.^[^ ^24^ ^]^ APOE is a glycoprotein involved in lipid transport and metabolism, serving as the brain's primary cholesterol transporter.^[^ ^25^ ^]^ It contributes to the pathogenesis of various neurodegenerative diseases through mechanisms such as amyloid‐beta (Aβ) deposition, tangle formation, oxidative stress, lipid homeostasis deregulation, synaptic plasticity loss, and cholinergic dysfunction.^[^ ^26^ ^]^ In the brain, APOE is primarily synthesized de novo, with a limited exchange between blood and brain APOE, leading to region‐specific expression of APOE in CNS diseases.^[^ ^27^, ^28^ ^]^ Clinical studies have confirmed that APOE expression in the cerebrospinal fluid (CSF), serum, or brain tissue is closely related to seizures or complications in patients with epilepsy.^[^ ^29^, ^30^ ^]^ These findings suggest APOE as a potential target for epilepsy diagnosis and treatment; however, research into its underlying mechanisms remains insufficient.

Under normal conditions, astrocytes are the primary APOE carriers,^[^ ^9^ ^]^ which is consistent with our scRNA‐seq analysis. In cell culture models, hyperactive neurons have been shown to release APOE‐associated fatty acids, which are subsequently absorbed by astrocytes to mitigate lipotoxic stress.^[^ ^31^ ^]^ Furthermore, Chen et al. identified a subset of *APOE*‐expressing LARA in both patients with TLE and epilepsy mouse models.^[^ ^10^ ^]^ *APOE* gene knockout reduced LARA formation and epileptic seizure activity in the mouse models. Although astrocytes are the predominant producers of APOE in the CNS, microglia increase their APOE expression during neurodegenerative processes.^[^ ^32^, ^33^ ^]^ Zou et.al identified a subset of *APOE*‐overexpressing spinal microglia in mice subjected to spared nerve injury.^[^ ^34^ ^]^ These cells removed inhibitory presynaptic terminals, disinhibiting PKCγ interneurons and enhancing transmission from low‐threshold Aβ fibers, thereby precipitating mechanical allodynia. *APOE* deficiency abolished the neuropathic pain‐induced increase in microglial synaptic engulfment and reversed glycinergic synapse loss. Notably, this mechanism was observed in both females and males, indicating sex generalizability. These findings suggest that APOE is not only an inflammatory marker but a functional regulator that modulates neuronal electrophysiology. Our study revealed that in the hippocampi of chronic epilepsy mouse models (14 days post‐induction), microglia became the primary carriers of elevated APOE expression, whether originating from microglia, astrocytes, hyperexcitable neurons, or peripheral sources. Through its active involvement in microglial differentiation and other processes, APOE influences epilepsy progression in multiple ways.

Studies in patients with Alzheimer's disease and animal models suggest that APOE can alter the microglial transcriptome, affecting immune responses, lipid metabolism, IFN signaling, and other functions.^[^ ^35^, ^36^ ^]^ Our pseudotime analysis indicates that under epileptic conditions, *APOE* is the main driver of microglial differentiation. Epilepsy is closely linked to inflammatory and immune responses. Tissue samples from patients with TLE‐HS showed significantly elevated cytokines, including CXCL8, IL‐1β, IL‐6, and TNF‐α.^[^ ^37^ ^]^ Moreover, our in vitro experiments demonstrated that APOE‐overexpressing microglia secrete TNF‐α, IL‐1β, and IL‐6 at levels comparable with those observed in microglia under simulated epileptic conditions. Elevated levels of TNF‐α not only directly increase neuronal firing rates but also indirectly affect synaptic plasticity, thereby promoting the “re‐wiring” of neural circuits into hyperexcitable pathways.^[^ ^21^ ^]^ IL‐1β can weaken GABA‐mediated inhibition, making neural networks more prone to excessive synchronous discharges.^[^ ^21^ ^]^ Meanwhile, under inflammatory conditions, IL‐6 becomes overactivated, inducing apoptosis‐related gene expression while concurrently suppressing axonal growth and survival gene expression, ultimately leading to axonal loss.^[^ ^21^ ^]^ This neuroinflammation can both trigger and exacerbate various epileptic seizure types through inflammatory cytokine release that further enhances neuronal excitability.^[^ ^18^ ^]^

Our combined bioinformatic analyses and cellular experiments confirmed several functional sites associated with *APOE*‐overexpressing microglia‐induced inflammatory response, namely the TLR4 and cyclic guanosine monophosphate‐adenosine monophosphate (cGAMP) synthase (cGAS)‐stimulator of IFN gene (STING) pathways. TLR4, primarily expressed in myeloid immune cells, triggers two signaling cascades upon activation: the myeloid differentiation primary response 88 (MyD88)‐dependent pathway (which induces pro‐inflammatory cytokine production) and the Toll/interleukin 1 receptor domain‐containing adapter inducing IFN‐β (TRIF)‐dependent pathway (which induces type I IFN and chemokine production).^[^ ^38^, ^39^ ^]^ Numerous studies have shown that inflammation‐mediated molecules can elicit neurotoxic effects by activating TLR4, thereby intensifying both the magnitude and spread of abnormal network discharges.^[^ ^21^, ^40^ ^]^ Inhibiting TLR4 may alleviate epileptic seizure frequency and duration, thereby offering a potential therapeutic avenue for drug‐resistant epilepsy. cGAS recognizes intracellular DNA and activates the STING pathway, leading to cGAMP production, which subsequently induces type I IFN and inflammatory cytokine expression. Animal studies have demonstrated that cGAS activation can drive microglia into an aging‐associated activated state, affecting neuronal survival through inflammatory cytokine secretion, particularly TNF.^[^ ^41^ ^]^ Inhibiting the cGAS‐STING pathway can alleviate hippocampal neuronal apoptosis and slow epilepsy progression.^[^ ^42^ ^]^ However, the current research on the relationship between the cGAS‐STING pathway and neuronal excitability remains limited.

Additionally, our findings in this study suggest that *APOE* overexpression in microglia may significantly alter their interactions with neighboring neurons and astrocytes. Reactive microglia not only contribute to neuronal loss and astrogliosis but also markedly influence neuronal excitability. Studies have demonstrated that in the hippocampus of patients with new‐onset refractory SE (NORSE) and TLE, microglial infiltration is increased, with the ratio of excitatory to GABAergic neurons positively correlating with microglial density.^[^ ^43^ ^]^ Moreover, microglia preferentially prune inhibitory synapses in epileptic brains.^[^ ^24^ ^]^ Under epileptic conditions, GABA released from inhibitory neurons may function as a “find‐me” cue that activates microglia and recruits them to inhibitory synapses. In response to GABA, microglia selectively engulf inhibitory (GABAergic) synapses, exacerbating the excitatory‐inhibitory imbalance of neural circuits and thereby promoting epileptogenesis and disease progression.^[^ ^44^ ^]^ Our in vivo and in vitro experiments further revealed that *APOE* knockout not only reduces hippocampal neuronal apoptosis and astrogliosis but also significantly decreases the excitability of pyramidal neurons and seizure frequency in TLE mice. Conversely, *APOE* overexpression in hippocampal microglia tends to increase neuronal excitability and exacerbate epileptic pathologies. Spatially, *APOE*‐overexpressing microglia predominantly interact within the CA1 and CA3 regions, areas most severely affected in both patients with TLE and epileptic mice.^[^ ^17^, ^43^ ^]^ Metabolic coupling between microglia and neurons was also significantly enhanced during TLE.

During epilepsy, hippocampal tissue metabolites are abnormally upregulated.^[^ ^45^, ^46^ ^]^ APOE, the brain's principal lipid carrier, facilitates the transport of cholesterol and other lipids among various neural cell types. In our study, microglia overexpressing *APOE* exhibited a more pronounced lipid metabolism reprogramming phenotype. Moreover, *APOE* significantly modulated glycerophospholipid metabolism in the hippocampus of TLE mice, affecting the synthesis and degradation of PS, PG, PA, CL, and its derivative MLCL. Notably, fresh hippocampal specimens from patients with TLE revealed marked disturbances in glycerophospholipid metabolism, particularly in the upregulation of PS and PG. These findings align with our metabolomics data, suggesting that *APOE* likely regulates cell membrane stability and signal transduction through these metabolic pathways, thereby promoting seizures.^[^ ^46^ ^]^ PS is mainly located on the inner leaflet of the cell membrane and is one of the brain's most abundant anionic phospholipids, playing key roles in membrane signal transduction, synaptic function, and immune signaling.^[^ ^47^ ^]^ However, under conditions of stress or apoptosis, PS can become externalized, thereby contributing to microglial‐mediated neuronal apoptosis.^[^ ^48^ ^]^ Furthermore, disrupting PS distribution can impair neurotransmitter transmission and synaptic plasticity.^[^ ^48^ ^]^ PG serves as a critical precursor for CL synthesis. Abnormal changes in PG levels may compromise the integrity of the inner mitochondrial membrane, thereby affecting adenosine triphosphate (ATP) synthesis and triggering oxidative stress.^[^ ^49^ ^]^ Additionally, PG regulates cell membrane fluidity and ion channel distribution, both of which are crucial for maintaining neuronal excitability and ensuring efficient synaptic transmission.^[^ ^50^ ^]^ PA, a key metabolic precursor and signaling lipid, regulates membrane curvature and modulates key cellular pathways such as the mammalian target of rapamycin (mTOR) and autophagy. In a study by Rodriguez de Turco et al., deletion of the *DGKε* gene, which selectively phosphorylates arachidonoyldiacylglycerol (20:4‐DAG) to form PA, conferred increased resistance to electroconvulsive shock in mice, as demonstrated by shorter tonic seizures and faster recovery^[^ ^51^ ^]^ CL, a *tetra*‐acyl phospholipid unique to the mitochondrial inner membrane, is essential for maintaining respiratory chain super‐complexes and cellular energy metabolism. The link between epileptic seizures and CL metabolic dysregulation has been demonstrated in both animal models and patients with epilepsy.^[^ ^45^, ^52^ ^]^ Elevated levels of anti‐CL antibodies indicate detrimental CL exposure during apoptosis or membrane remodeling under epileptic conditions.^[^ ^52^ ^]^ MLCL, generated when CL loses one acyl chain, typically arises during CL remodeling or degradation. Direct studies on MLCL in epilepsy are limited; however, its accumulation may further impair mitochondrial membrane integrity and compromise enzymatic complex function, thereby exacerbate energy metabolism deficits and perpetuating the vicious cycle of epilepsy. Overall, these findings underscore the important role of *APOE* in modulating hippocampal metabolism during epilepsy, further complementing our understanding of the molecular mechanisms underlying the ketogenic diet's effects on TLE.

Our study explored the effects of microglial *APOE* overexpression and *APOE* on TLE from multiple perspectives; however, some limitations should be acknowledged. First, to minimize tissue heterogeneity, the single‐cell analyses were conducted on mouse samples rather than on human specimens, and the cell suspensions were not nuclei‐enriched, resulting in a low neuronal yield and potentially reducing data reliability. Second, the pathways identified in the phenotypic experiments were not validated longitudinally. Third, given the limited yield and viability of primary microglia, the poor transduction efficiency of microglia with AAV vectors, their rapid phenotypic alterations ex vivo, and extensive cell death induced by the cytotoxic effects of KA stimulation, we employed cell lines for our in vitro experiments. Fourth, as the primary focus of our study was not on lipid metabolic disturbances in the hippocampus, the metabolomics and lipidomics results were not further validated through additional animal or cellular experiments. Finally, because *APOE* expression in microglia is naturally low under physiological conditions, generating microglia‐specific *APOE* knockout mice is challenging and may not fully suppress the gradual increase in APOE observed in TLE. Consequently, some experiments, including the metabolomics analyses, did not achieve a targeted microglial knockdown, which may have compromised the specificity of our results. Future studies, subject to appropriate ethical approvals, should aim to acquire human tissue samples and further investigate the specific role of microglial APOE expression using both in vitro and in vivo models, thus enabling a more comprehensive understanding of the present findings.

## 4 Conclusion

Our study comprehensively explored the role of APOE in the hippocampus during TLE, with a particular emphasis on its overexpression in microglia. Our findings demonstrate significant microglial *APOE*‐driven alterations in microglial states, neuroinflammatory activation, neuronal injury, gliosis, neuronal excitability, and metabolic dysregulation in the hippocampus. These findings strongly indicate that *APOE*, especially microglial *APOE*, actively contributes to the progression of TLE and HS, highlighting potential therapeutic targets and avenues for future epilepsy treatments.

## 5 Experimental Section

### Construction of the Epilepsy Model

Male C57BL/6J and *APOE* ^−/−^ mice (aged 6–8 weeks, weighing 24–26 g) were acquired from GemPharmatech Co., Ltd, Nanjing, China. The TLE model was established via stereotactic injection of KA into the hippocampus (Figure S5, Supporting Information). Each mouse was weighed, anesthetized, and securely fixed in a stereotaxic frame. The skull was exposed to locate the hippocampus (CA1) using coordinates from the mouse brain atlas (AP: −2.0, ML: 1.5, DV: −2.0). Precision drilling at these coordinates was conducted using a 0.5 mm skull drill. KA (10 mg mL^−1^, #ab120100, Abcam, United States) was administered intrahippocampally using a Hamilton syringe attached to a Quintessential Stereotaxic Injector (Stoelting), injecting 200 nL over 1 min. The control group received PBS. The needle remained in place for an additional 2 min post‐injection to prevent backflow.^[^ ^53^ ^]^ The incision was sealed with Vetbond dermal glue (3M), and mice were placed in a warmed recovery chamber. Seizures were monitored and scored using a modified Racine scale for 2 h post‐injection. Intrahippocampal KA injection induced convulsive SE; therefore, the seizures were behaviorally quantified using a modified Racine scale: I) continuous ear and facial twitching with whisker trembling; II) head and neck jerks (head nodding); III) forelimb clonus with rearing; IV) forelimb clonus accompanied by multi‑limb twitching; V) generalized tonic–clonic seizures with loss of balance and falling down.^[^ ^54^ ^]^ The success of the epilepsy model was defined by the occurrence of three consecutive stage IV‐V seizures within the observation period. After injection, animals were housed under a 12‐h light‐dark cycle (light: 8:00–20:00) in a controlled environment (22±1 °C and 50–60% humidity) with free access to food and water.

The latent period from the initial cerebral insult to chronic epilepsy development (range: 2–14 days) in this model.^[^ ^17^ ^]^ Therefore, mice were sacrificed at different time points in 2 weeks (all experienced spontaneous recurrent seizures within 1 week before sacrifice), with rapid extraction of whole brains and hippocampi. Whole brains were paraffin‐embedded, while hippocampi were flash‐frozen in liquid nitrogen. Subsequently, all samples were stored at −80 °C for future experiments. All animal experiments were conducted in accordance with National Institutes of Health guidelines and were approved by the Animal Ethics Committee of Xuanwu Hospital, Capital Medical University (LYS [2021]118).

### Human Sample Collection

Patients diagnosed with TLE were recruited from Xuanwu Hospital. Comprehensive preoperative evaluations, including detailed medical history, neurological examinations, electrophysiological studies, and neuroimaging, confirmed the diagnosis. All patients underwent anterior temporal lobectomy with or without amygdalohippocampectomy as part of their clinical treatment. The resected hippocampal tissues were immediately sent to the Department of Pathology for histopathological examination to assess the presence or absence of HS. Based on the histopathological findings, the 12 hippocampal tissues were categorized into two groups: TLE with HS and TLE without HS. The collected hippocampal tissues from both groups were promptly frozen in liquid nitrogen and stored at −80 °C until further analysis. Clinical and pathological information of the participants is listed in Table S2 and Figure S9 (Supporting Information). This study was approved by the Ethics Committee of Xuanwu Hospital, Capital Medical University, China (No. 20191102). Written informed consent was obtained from all participants or their legal guardians before sample collection. All procedures adhered to the ethical standards of the Declaration of Helsinki.

### Public Data Extraction

Public data were extracted from Series Matrix Files of GSE73878 and GSE71058 in the GEO database (https://www.ncbi.nlm.nih.gov/geo/). Protein secretion gene set scores in tissue samples were computed using the single‐sample GSEA (ssGSEA) algorithm implemented in the GSVA R package (v1.52.3).

### Tissue Dissociation and Cell Purification

The hippocampal tissue was placed in a sterile dish containing 10 mL of 1X Dulbecco's PBS (DPBS, #14190144, Thermo Fisher) on ice to remove any remaining storage solution, followed by mincing. Tissue digestion was performed using 0.25% Trypsin (#25200‐072, Thermo Fisher) and 10 µg mL^−1^ deoxyribonuclease I (#11284932001, Sigma) in PBS with 5% fetal bovine serum at 37 °C, with shaking at 50 rpm for ≈40 min to ensure dissociation, with intermittent cell harvesting at 20‐min intervals to enhance yield and viability. Subsequently, the cell suspension was filtered through a 40‐µm nylon filter, and red blood cells were lysed using a 1X Red Blood Cell Lysis Solution (#00‐4333‐57, Thermo Fisher). After washing with DPBS (2% FBS), the cell viability was assessed using 0.4% Trypan blue on a Countess II Automated Cell Counter.

### 10x Library Preparation and Sequencing

Cells and beads carrying unique molecular identifiers (UMIs) and cell barcodes were combined to near saturation in Gel Beads‐in‐emulsion (GEMs) for 10x library preparation. Cell lysis released polyadenylated RNAs, which hybridized to the beads that underwent reverse transcription, tagging each complementary DNA (cDNA) at its 5’ end with UMIs and cell labels. This process included second‐strand cDNA synthesis, adaptor ligation, and amplification. Sequencing libraries focused on 3’ transcript ends and followed the Chromium Single Cell 3ʹ v3.1 protocol. Libraries were quantified using a High‐Sensitivity DNA Chip (Agilent Technologies) on a Bioanalyzer 2100 and the Qubit High‐Sensitivity DNA Assay (Thermo Fisher Scientific). Sequencing was performed on Illumina Xplus using a 2 × 150 chemistry.

### Single‐Cell RNA Sequencing Data Processing

scRNA‐seq was conducted at Shanghai Majorbio Bio‐pharm Biotechnology Co., Ltd. (Shanghai, China), following the manufacturer's instructions (Illumina, San Diego, CA, USA). Using Cell Ranger (v7.1.0), reads were processed and aligned to the mouse GRCm38 genome via spliced transcripts alignment to a reference (STAR), and UMI counting was performed to generate gene‐barcode matrices, filtering out non‐cell barcodes. After integration into Seurat (v4.1.1) for quality control, the cells were filtered based on transcript counts, gene counts, and mitochondrial gene read percentages within predefined thresholds. Data were normalized, and variable genes were identified, followed by data integration across samples using “anchors.” Subsequently, PCA was applied to the top 30 components, followed by Uniform Manifold Approximation and Projection (UMAP) for 2D cluster visualization. Cell clustering was executed via graph‐based clustering on PCA‐reduced data using the Louvain method, following the construction of a shared nearest‐neighbor graph. Subclustering utilized the same scaling, dimensionality reduction, and clustering approach (UMAP) on specific data subsets, often confined to one cell type. Significant DEGs in each cluster compared with others were identified using the Wilcoxon Rank‐Sum Test, and cell types were determined using SingleR and known marker genes.^[^ ^55^ ^]^

### Differentially Expressed Genes Enrichment Analysis

Enrichment analyses were performed using the DAVID database (https://david.ncifcrf.gov/) to assess GO terms (biological process, molecular function, and cellular component) and KEGG pathways among the differentially expressed genes. Enrichment of genes/transcripts in the gene sets was evaluated using Fisher's exact test. *P*‐values were adjusted for multiple testing using the Benjamini–Hochberg method, and adjusted *p*‐values < 0.05 were considered statistically significant.

### Pseudotime and Cell–Cell Interaction Analysis

Microglia were isolated for pseudotime analysis. VECTOR was employed to infer developmental trajectories in UMAP space, using the vector.autoCenter function to identify the developmental root.^[^ ^56^ ^]^ This root state was subsequently designated as the starting point for Monocle3 trajectory reconstruction and cell ordering.

### Spatial RNA Sequencing

The tissue was gently washed with cold PBS, cut into 4–6 mm^3^ pieces, and placed cut side down into a plastic optimal cutting temperature‐filled mold. The mold was snap‐frozen in chilled isopentane. The sample was then sent to Majorbio Bio‐Pharm Technology Co., Ltd. (Shanghai, China) for S1000 spatial RNA sequencing. Additionally, permeabilization and library construction were performed according to user guidelines. The Illumina library was sequenced using Illumina NavoSeq. The raw data from Illumina were mapped to the *Mus musculus* reference genome using BSTMatrix v2.3.0, with default parameters. Image alignment and adjustment were performed in BSTViewer, and the corresponding level 6 (42 µm) spatial matrix was used for downstream analyses. The UMAP and clustering information were obtained using an R script (Bmk Space mapping R, http://www.bmkmanu.com/archives/513) based on Seurat 4.3.0 (39) provided by the manufacturer (parameters: nCount_Spatial>100, min.cells = 0, min.features = 0, dims = 1:30, resolution = 1.5). Microglia and immune‐related signature scoring were performed using the AddModuleScore function in Seurat with default parameters.

### Spatial Map of Cell Dependencies

MISTy implemented in mistyR (v1.12.0), was employed to quantify the extent to which the abundance of each major cell type predicts the presence of others.^[^ ^57^ ^]^ Using an intrinsic perspective, correlations were evaluated among RCTD deconvolution estimates within defined spatial regions. Using MISTy's intrinsic view and Paraview, standardized importance scores were calculated for each predictor‐response cell type pair and summarized these by their median across regions. These median standardized importance values were interpreted as measures of intercellular dependency in a colocalized context.

### Western Blot

Hippocampal samples from mice were lysed in buffer (100 mm NaCl, 50 mm *Tris*‐HCl, 0.5% NP‐40) with 1× concentration of both protease and phosphatase inhibitors (Thermo Fisher, 78440). Immunoprecipitation was performed overnight at 4 °C using IgG or antigen‐specific antibodies attached to Dynabeads Protein G (Thermo Fisher, 10004D). For standard western blot (WB) analysis and capillary electrophoresis, cellular lysates were prepared using Laemmli buffer (Bio‐Rad) supplemented with the same inhibitor cocktail (Thermo Fisher, 78440). Electrophoresis was conducted on a 4–12% sodium dodecyl sulfate‐polyacrylamide gel (Invitrogen, NP0321BOX), and proteins were transferred onto polyvinylidene fluoride membranes. Subsequently, the membranes were blocked using 5% bovine serum albumin (BSA) in tris‐buffered saline with 0.1% Tween 20 before incubation with specific antibodies. WB images were captured using an Azure (C300) imaging system (Azure Biosystems). Capillary electrophoresis was performed using the Jess system (JS4929), following the protocols provided by ProteinSimple (SM‐W004, San Jose, CA). The primary antibodies used were APOE (#66830‐1‐Ig, 1:3000, Proteintech), TLR4 (#19811‐1‐AP, 1:1000, Proteintech), cGAS (#29958‐1‐AP, 1:1000, Proteintech), STING (#19851‐1‐AP, 1:1000, Proteintech), and β‐actin (#66009‐1‐Ig, 1:5000, Proteintech). Subsequently, sections were incubated with a horseradish peroxidase conjugate specific to the corresponding species.

### Immunofluorescence Analysis

All immunofluorescence procedures were performed with mild agitation at room temperature. Tissue slices underwent three washes in PBS for 10 min each, followed by overnight incubation with the primary antibodies. These antibodies were diluted in PBS with 0.3% Triton X‐100 and 5% normal serum (either goat or donkey, depending on the secondary antibody species), except when using the Mouse‐on‐Mouse Fluorescein kit. Following primary antibody incubation, slices underwent three additional PBS washes, followed by a two‐hour dark incubation with the secondary antibody solution prepared in PBS with 0.3% Triton X‐100, except when using the Mouse‐on‐Mouse kit. Subsequently, the slices were briefly rinsed in PBS for 5 min and stained using the 4’,6‐diamidino‐2‐phenylindole nuclear marker (# D1306, 1:10000, Molecular Probes) for 5 min, and subjected to a final PBS rinse. Finally, the slices were mounted on gel‐coated slides, cover slipped with Prolong gold anti‐fade (# P36930; Vector Laboratories), and stored at 4 °C until further analysis. The primary antibodies were used as follows: used against Iba1 (#ab5076, 1:500, Abcam), NeuN (#ab177487, 1:400, Abcam), GFAP (#ab7260, 1:500, Abcam), APOE (#66830‐1‐Ig, 1:400, Proteintech), and DAPI (#S7113, 1:1,000, Sigma–Aldrich).

Hippocampal sections were imaged on a Pannoramic MIDI slide scanner (3DHISTECH, Hungary) equipped with a Plan‐Apochromat 20× objective lens, a 0.63× camera adapter, and a Point Grey GS3‐U3‐51S5M‐C camera. This configuration yielded an effective pixel size of ≈0.27 µm per pixel (calculated from the camera pixel size, 3.45 µm, divided by the nominal optical magnification: 3.45 µm/(20×0.63) ≈ 0.274 µm per pixel). Fluorescent images were viewed and exported using CaseViewer 2.3 (3DHISTECH, Hungary). Immunofluorescence image quantification was performed using ImageJ2 (v2.3.0), an ImgLib2‐based, N‐dimensional rewrite of ImageJ that decouples processing from the GUI while maintaining backward compatibility with ImageJ 1.x.

### Enzyme‐Linked Immunosorbent Assay for Inflammatory Cytokine Determination

A BV2 microglial cell line stably overexpressing *APOE* (BV2‐OE, vector information: pPB [Exp]‐EF1A‐EGFP>CAG‐mApoe [NM_009696.4]>PGK‐Puro) was generated. Details of the construction and validation of the *APOE*‐overexpressing BV2 cell line are provided in Table S3 and Figure S10 (Supporting Information). BV2 cells, BV2‐OE cells, and HT22 neurons were cultured on glass coverslips. In the HT22 neuron culture dishes, 100 µm KA or PBS was added, and after 24 h of treatment, the conditioned medium was collected. After removing the culture medium from BV2 and BV2‐OE cells, the conditioned medium from KA‐ or PBS‐treated HT22 cells was added, and the cells were incubated at 37 °C for 48 h. Additionally, an inhibitor treatment group was established by diluting the Anti‐Mouse APOE Monoclonal Antibody (Clone HJ6.3, #RMB98601, AntibodySystem SAS, Paris, France) in conditioned medium from HT22 cells to a final concentration of 10 µg mL^−1^, and adding it to the corresponding BV2 cell culture dishes. The target of HJ6.3 was the mature APOE protein, whether secreted into the extracellular space or displayed on the cell surface. Its variable region specifically recognizes and binds APOE, sterically blocking interactions between APOE and its receptors or pathological ligands. Subsequently, the cells and conditioned media were collected. BV2 and BV2‐OE cells were washed three times with pre‐cooled PBS, lysed with radioimmunoprecipitation assay buffer containing protease and phosphatase inhibitors, and the lysates were collected for WB analysis. The final conditioned media from BV2 and BV2‐OE cells were collected, filtered through a 0.22 µm membrane, and kept on ice for cytokine detection. According to the instructions of the ELISA kits, TNF‐α (#EM0183, FineTest, Wuhan, China), IL‐1β (#EM0109, FineTest, Wuhan, China), and IL‐6 (#EM0121, FineTest, Wuhan, China) levels in the conditioned media were measured.

### In Vivo Electroencephalogram Recording

Epilepsy models were established in eight‐week‐old male C57BL/6J and *APOE* ^−/−^ mice following previously described methods. Information and validation of the *APOE* ^−/−^ mice are provided in Figures S11 and S12 (Supporting Information). After a three‐day acclimation period, the mice were anesthetized with pentobarbital sodium (80 mg kg^−1^, intraperitoneally) and positioned in a stereotaxic apparatus. After skull exposure, surface electrodes (Model number: XJ230912X, Kedou Brain‐Computer Technology, Suzhou, China) were implanted over the frontoparietal cortex and secured with dental cement. Postoperatively, the mice were allowed to recover for 1 week in their home cages. Subsequently, the electrodes were connected to a data acquisition system (Neuracle, Changzhou, China) via electrode wires, enabling continuous EEG recording for 3 days. Specifically, intracranial EEG monitoring was performed using a high‐frequency, high‐density digital electroencephalograph (Model: NSHR, Neuracle, Changzhou, China) in conjunction with the manufacturer's EEGRecorder software (v1.0.0.9306). Seizure quantification included spontaneous recurrent seizures, encompassing both focal electrographic seizures and generalized tonic–clonic seizures. Seizures were manually counted by trained technicians who reviewed continuous synchronized video‐EEG recordings in a blinded manner, applying electrographic thresholds to distinguish seizure events from baseline activity and ensure electroclinical correlation.^[^ ^58^, ^59^ ^]^ Seizure severity was graded using the modified Racine scale as described previously. Intracranial EEG data were analyzed using in‐house scripts in MATLAB R2016b (The MathWorks, Inc., Natick, MA, USA). The power spectral density and time–frequency analysis code have been deposited on Zenodo (https://www.zenodo.org: https://doi.org/10.5281/zenodo.15882208).

### Adeno‐Associated Virus‐Mediated APOE Overexpression in Microglia

The mice underwent intracerebroventricular injections targeting the dorsal hippocampal region. The AAVs (2 µL for each hemisphere) were injected into the mouse dorsal hippocampal region as mentioned above. Experimental groups included: i) AAV‐CD68‐eGFP‐NC‐MG1.2 control virus at a concentration of 2.06 × 10^13^ viral genomes per milliliter (VG mL^−1^) and ii) APOE (*M. musculus*)‐MG1.2 virus (8.50 × 10^12^ VG mL^−1^). The microglia‐specific AAV‐MG1.2, as well as the plasmids encoding APOE and control sequences, were designed, constructed, and packaged by GenePharma Co., Ltd. (Suzhou, China). Details of AAV vector construction for the microglial targeting strategy are provided in Table S4 (Supporting Information). AAV transduction efficiency and the efficacy of APOE overexpression are presented in Figures S12 and S13 (Supporting Information).

### Whole‐Cell Patch‐Clamp Recording of Action Potentials and Spontaneous Excitatory Postsynaptic Currents in CA3 Neurons

Acute hippocampal slices (300 µm) were prepared in oxygenated ice‐cold cutting solution (in mm: 110 sucrose, 62.5 NaCl, 2.5 KCl, 1.25 NaH_2_PO_4_, 25 NaHCO_3_, 0.5 CaCl_2_, 7 MgCl_2_, 10 glucose) and recovered in artificial CSF (32 °C, ≥1 hr). CA3 pyramidal neurons were identified using infrared differential interference contrast microscopy (Olympus BX51WI) based on somatic morphology and anatomical landmarks. Current‐clamp recordings (K‐gluconate internal solution, 3–5 MΩ electrodes) employed depolarizing steps (0–210 pA, 30 pA increments, 500 ms) to quantify AP frequency. Voltage‐clamp sEPSCs (Cs‐methanesulfonate internal solution, tetradotoxin 1 µM, V = −70 mV) were recorded at 10 kHz (2 kHz low‐pass) and analyzed using MiniAnalysis 6.0.3 (threshold: 5 pA, rise time <2 ms) for frequency and amplitude. Neurons with series resistance >20 MΩ or drift >20% were excluded.

### Sample preparation for liquid chromatography‐tandem mass spectrometry analysis

The hippocampus (injection side) of *APOE*‐KO and WT epileptic model mice (*n* = 6 per group) was extracted post‐perfusion. For metabolomic analysis, ≈50 mg of tissue was placed in a 2 mL centrifuge tube with a 6 mm grinding bead. Metabolites were extracted using 400 µL methanol: water (4:1, v:v, Thermo Fisher Scientific) containing 0.02 mg mL^−1^ L‐2‐chlorophenylalanine (internal standard). Samples were ground using a Wonbio‐96c tissue grinder (Shanghai Wanbo Biotechnology Co., Ltd) at −10 °C and 50 Hz for 6 min, followed by ultrasonication at 5 °C and 40 kHz for 30 min. The samples were incubated at −20 °C for 30 min before centrifugation (13 000 g, 15 min, 4 °C). Subsequently, the supernatant was transferred to an autosampler vial for analysis.

For lipidomic analysis, ≈50 mg of tissue was similarly processed. Lipids were extracted using 280 µL methanol: water (2:5, v:v), followed by 400 µL methyl *tert*‐butyl ether (MTBE, Adamas‐beta, China). The mixture was ground (−10 °C, 50 Hz, 6 min), ultrasonicated (5 °C, 40 kHz, 30 min), and incubated at −20 °C for 30 min. After centrifugation (13 000 g, 15 min, 4 °C), ≈350 µL of the supernatant was dried using a JXDC‐20 nitrogen evaporator (Shanghai Jingxin, China). The dried residue was re‐dissolved in 100 µL isopropanol: acetonitrile (1:1, v:v, Thermo Fisher Scientific), vortexed (30 s), ultrasonicated (5 min, 40 kHz), and centrifuged (13 000 g, 10 min, 4 °C) before transfer to an ultra‐high performance liquid chromatography‐tandem mass spectrometry (UHPLC‐MS/MS) system.

### Metabolomics and Lipidomics Analysis

LC‐MS/MS‐based metabolomics and lipidomic analyses were performed using a UHPLC‐Q Exactive HF‐X system (Thermo Fisher Scientific, USA). For metabolomics, LC‐MS raw data were processed using Progenesis QI (Waters Corporation, Milford, MA, USA), including baseline correction, peak detection, integration, retention time correction, and alignment. Metabolites were identified by matching the mass spectrometry (MS) and tandem mass spectrometry (MS/MS) spectra with the HMDB (http://www.hmdb.ca/) and Metlin (https://metlin.scripps.edu/) databases. For lipidomics, LC‐MS data were analyzed using LipidSearch (Thermo, CA, USA), and lipid features were matched against an in‐house database (mass error <10 ppm). Lipid identities were confirmed based on the MS/MS matching scores.

Variables detected in at least 80% of samples within one group were retained. Missing values were imputed using the minimum observed value, total intensities were normalized, variables with quality control relative standard deviation (QC RSD) >30% were removed, and data were log_10_‐transformed. PCA and orthogonal PLS‐DA were performed using the R ropls package with seven‐fold cross‐validation. Differential metabolites were identified based on VIP scores (>1) and Student's t‐test (*p* < 0.05). Pathway enrichment analysis was conducted using the KEGG database (https://www.kegg.jp/kegg/pathway.html) and Fisher's exact test.

### Drug Prediction

The 3D structures of the proteins were obtained from the UniProt database (https://www.uniprot.org/). First, the ligand's SDF structure was retrieved from the PubChem database (https://pubchem.ncbi.nlm.nih.gov/) and converted into a protein databank file using OpenBabel. Second, AutoDock (Version 1.5.6) was used to remove water molecules, add hydrogen atoms to the protein target, and convert both the active compound and target protein into pdbqt format. Subsequently, molecular docking was performed using AutoDock. Finally, the protein‐ligand complexes were visualized in 3D and 2D using PyMOL (version 3.1) and LigPlot (version 2.2).

### Statistical Analysis

For normally distributed data, the independent samples t‐test was used to compare the means of the two independent sample groups. For non‐normally distributed data or small sample sizes that did not meet normality assumptions, the non‐parametric Mann–Whitney U test was applied. One‑way and two‑way ANOVAs were also performed for multi‑group comparisons (reported as F (df_1_, df_2_) = value). Cohen's d was calculated for all t‑tests and partial η^2^ for all ANOVAs as standardized effect‑size measures. Significance was set at p < 0.05, and all values are expressed as means ± standard deviation (SD). All experiments were performed in triplicate. For all quantitative assays (human or mouse tissue analyses, immunofluorescence, and in vivo EEG), sample sizes were at least *n* = 6 per group. Sample size was guided by precedent from previous similar studies,^[^ ^10^ ^]^ resource‑equation calculations,^[^ ^60^ ^]^ and simplified ANOVA‑based formulas.^[^ ^61^ ^]^ Data analysis and graphing were performed using R (version 4.4.1) and GraphPad Prism 8 (GraphPad Software, Inc.). Detailed statistical outputs, including standardized effect sizes, degrees of freedom, and p‑values, are provided in Table S5 (Supporting Information).

### Ethics Approval and Consent to Participate

The human subjects involved in this study were recruited in accordance with the Declaration of Helsinki, and the study was approved by the local ethics committee of Xuanwu Hospital, Capital Medical University, Beijing, China (No. 20191102). Written informed consent was obtained from all participants (or their legal guardians, in the case of minors). Animal experiments were approved by the Animal Ethics Committee of Xuanwu Hospital, Capital Medical University (LYS [2021]118).

## Conflict of Interest

The authors declare no conflict of interest.

## Author Contributions

J.S., Z.L., and X.S. contributed equally to this work. G.Z., Y.S., J.S., and J.X. designed the study. J.S., Z.L., and X.S. wrote the manuscript and conducted the analyses. X.S., Y.L., Y.Y., and J.X. provided the experimental guidance. J.S. and X.S. conducted the experiments. Y.Y., Z.S., L.J., and H.D. assisted in the collection of samples and data. G.Z., Y.L., and J.X. provided advice on the discussion. The manuscript was completed under the supervision of G.Z. and Y.S. All authors have read and approved the final manuscript.

## Supporting information



Supporting Information

## Data Availability

The data that support the findings of this study are available in the supplementary material of this article.
