# Supplementary material for: Elevated Apolipoprotein E Expression in Hippocampal Microglia Drives Temporal Lobe Epilepsy Progression
Source: Adv Sci (Weinh). 2025 Oct 14;13(2):e05778. doi: 10.1002/advs.202505778 (PMC12786283; doi:10.1002/advs.202505778)
Supplement: Supplementary file 1 — Supporting Information [file ADVS-13-e05778-s001.pdf]

WILEY-VCH

ADVANCED SCIENCE
Open Access

Supporting Information

Elevated Apolipoprotein E Expression in Hippocampal Microglia Drives Temporal Lobe Epilepsy Progression

Jianwei Shi[†], *Zesheng Li*[†], *Xin Sun*[†], *Yanfeng Yang, Yumin Luo, Ziang Song, Hengxin Dong, Lei Jin, Jing Xie*[*], *Yongzhi Shan*[*], *Guoguang Zhao*[*]

Affiliations:

J. Shi, Z. Li, Y. Yang, Y. Luo, Z. Song, H. Dong, L. Jin, Y. Shan, G. Zhao

Department of Neurosurgery, Xuanwu Hospital, Capital Medical University, No. 45 Changchun Road, 100053, Beijing, China.

China International Neuroscience Institute, No. 45 Changchun Road, 100053, Beijing, China.

Clinical Research Center for Epilepsy, Capital Medical University, No. 45 Changchun Road, 100053, Beijing, China.

X. Sun

Department of Pharmacology, Jiangsu Key Laboratory of Neurodegeneration, Nanjing Medical University, No. 101 Longmian Road, Nanjing, 211166, China

J. Xie

Deanery of Biomedical Sciences, Edinburgh Medical School, College of Medicine and Veterinary Medicine, University of Edinburgh, Edinburgh EH8 9AG, UK.

Jiangsu Botanical Medicine Refinement Engineering Research Center, Nanjing University of Chinese Medicine, Nanjing 210023, China.

† J. Shi, Z. Li, and X. Sun contributed equally to this work.

* Correspondence to G Zhao, Y Shan and J Xie.

Content

Tables

Table S1. Comparison of hippocampal *APOE* gene expression differences between groups based on single-cell transcriptomics……… **1**

Table S2. Demographic and clinical characteristics of study participants……… **3**

Table S3. Construction and validation details of BV2 cell lines overexpressing APOE……… **4**

Table S4. Details of microglia-targeted AAV vector construction ……… **5**

Table S5. Statistical analysis values for the main figures……… **6**

Figures

Figure S1. Base error rate distribution of the raw data (scRNA-seq quality control) ……… **16**

Figure S2. Base composition distribution plot (scRNA-seq quality control) ……… **16**

Figure S3. Bubble plot of marker gene expression……… **17**

Figure S4. Expression of inflammatory cytokines in neuron-conditioned medium……… **18**

Figure S5. Establishment of the TLE model (video) and status epilepticus severity post-induction……… **19**

Figure S6. Power spectral density (PSD) plot of four groups……… **21**

Figure S7. Video-EEG recordings from TLE mice (video) and quantification of chronic-phase seizure severity using the modified Racine scale………**22**

Figure S8. Metabolomics and lipidomics quality control……… **23**

Figure S9. Histopathological sections of hippocampal tissue in TLE patients……… **24**

Figure S10. Cell state, morphology and EGFP reporter expression of BV2 overexpressing APOE and BV-2 control (with qPCR validation) ……… **26**

Figure S11. Information and validation of *APOE*-KO mice……… **28**

Figure S12. APOE protein expression in hippocampus of *APOE*-KO, WT, and AAV-*APOE*-injected mice……… **29**

Figure S13. Immunofluorescence validation of microglia-targeted AAV overexpressing APOE infection……… **30**

Table S1. Comparison of hippocampal *APOE* Gene expression differences between groups based on single-cell transcriptomics.

Group	*APOE* Expression	Significance (Yes/No)
TLE vs NC (Overall)	UP	Yes
TLE_microglia vs NC_ microglia	UP	Yes
TLE_neuron vs NC_ neuron	UP [a#]	Yes
TLE_astrocyte vs NC_astrocyte	UP [b#]	No
TLE_ microglia vs others	UP	Yes
NC_ microglia vs others	DOWN [a#]	Yes
TLE_ neuron vs others	DOWN	Yes
NC_ neuron vs others	DOWN [a#]	Yes
TLE_astrocyte vs others	DOWN	Yes
NC_astrocyte vs others	UP [a#]	Yes

Description:

1. Microglia: Under normal conditions, microglia express APOE at low basal levels. However, in epilepsy, *APOE* expression in microglia is significantly elevated compared to both their normal state and other cell types. This suggests that microglia may be the primary contributors to the overall increase in *APOE* levels observed in epilepsy.

2. Neurons: Typically, neurons show little to no *APOE* expression under normal conditions. In the context of epilepsy, neuronal *APOE* expression increases notably compared to their baseline levels. However, relative to other cell types, this increase is less pronounced, indicating that neurons are likely not the main contributors to the overall rise in *APOE* levels during epilepsy.

3. Astrocytes: Under normal conditions, astrocytes are the primary producers of *APOE*. In epilepsy, *APOE* expression in astrocytes is elevated compared to their normal state, but this increase is less significant relative to other cell types. Therefore, astrocytes are unlikely to be the main source of the overall *APOE* elevation observed in epilepsy.

4. Conclusion: While both neurons and astrocytes show increased *APOE* expression in epilepsy compared to normal conditions, they do not appear to be the primary contributors to the overall rise in *APOE* levels. In contrast, microglia transition from low or non-existent *APOE* expression under normal conditions to high expression levels in epilepsy, surpassing those of other cell types. This suggests that microglia are likely the

main cellular source of the increased *APOE* observed in epilepsy.

Note:

1. Trends marked with # have been validated in other studies:

a#. Xu Q, Bernardo A, Walker D, Kanegawa T, Mahley RW, Huang Y. Profile and regulation of apolipoprotein E (ApoE) expression in the CNS in mice with targeting of green fluorescent protein gene to the ApoE locus. J Neurosci. 2006 May 10;26(19):4985-94. DOI: 10.1523/JNEUROSCI.5476-05.2006. PMID: 16687490; PMCID: PMC6674234.

b#. Chen ZP, Wang S, Zhao X, Fang W, Wang Z, Ye H, Wang MJ, Ke L, Huang T, Lv P, Jiang X, Zhang Q, Li L, Xie ST, Zhu JN, Hang C, Chen D, Liu X, Yan C. Lipid-accumulated reactive astrocytes promote disease progression in epilepsy. Nat Neurosci. 2023 Apr;26(4):542-554. DOI: 10.1038/s41593-023-01288-6. PMID: 36941428.

2. Differential expression between annotated cell groups was assessed using the Wilcoxon rank-sum test (Seurat-Find Markers). For each annotated cell type (e.g., neurons), expression was compared between sample groups (for example, TLE vs NC across all samples); comparisons of a single cell type versus all other cell types (e.g., TLE_microglia vs others) were performed with the same settings. Log2 fold changes were calculated from normalized expression counts to reduce spurious calls, and all tests were two-sided. Genes were considered significant when they met an absolute log2 fold change ≥ 0.25 and an adjusted P value < 0.05 (Benjamini–Hochberg correction). If the analysis returned only raw P-values, we applied Benjamini–Hochberg correction post hoc; reported significant genes therefore satisfy the stated fold-change threshold and multiple-testing adjusted significance criteria.

Table S2. Demographic and clinical characteristics of study participants.

No.	Gender (Male/Female)	Age at surgery (Years)	Mean seizures at baseline (Times/3 months)	Duration (Years)	TEL with HS (Yes/No)
1	Male	34	90	11	Yes
2	Male	47	24-36	15	No
3	Male	40	90	8	No
4	Male	62	100	30	Yes
5	Male	52	360	33	Yes
6	Male	44	180-270	8	No
7	Male	60	20-30	29	Yes
8	Male	32	90	9	No
9	Male	55	360-900	35	Yes
10	Male	34	270	17	Yes
11	Male	50	60	5	No
12	Male	27	9-12	21	No

Note: Study was approved by Xuanwu Hospital Ethics Committee. All participants provided written informed consent. Details presented in Experimental Section/Methods.

Table S3. Construction and validation details of BV2 cell lines overexpressing APOE

A. Vector constructs

Gene	Clone ID	Vector backbone and insert
Experimental	TSC911-1	pPB [Exp]-EF1A-EGFP>CAG-mApoe [NM_009696.4]>PGK-Puro
Control	PBC03	pPB [Exp]-EF1A-EGFP>CAG-[ORF-stuffer]>PGK-Puro

B. qPCR primer information

Gene	Primer Name	Sequence (5′→3′)	Amplicon Size
mGAPDH	mGAPDH-F	GTCAAGGCCGAGAATGGGAA	237 bp
	mGAPDH-R	CTCGTGGTTCACACCCATCA	
mApoe	mApoe -F	CAGATCAGCTCGAGTGGCAA	151 bp
	mApoe -R	TCCATCAGTGCCGTCAGTTC	

C. Relative quantification of Apoe expression by qPCR

Sample	mApoe ①	mGAPDH ②	ΔCT(①-②)	Relative Expression
BV-2#mApoe#Poolcells	15.76±0.01	15.64±0.07	0.12	2.61
BV-2#control#Poolcells	17.74±0.14	16.24±0.11	1.50	1.00

Note: All experimental groups exhibited efficient transfection. The mApoe gene expression in the experimental group was 2.61-fold that of the control group (see validation in Figure S10).

Table S4. Details of AAV vector construction for microglial targeting strategy.

Data Item	Details	Notes
Vector Backbone	pAAV-CD68	Driven by the CD68 promoter, which is highly specific to microglia according to literature
Promoter	CD68 promoter	A microglial marker gene promoter ensuring transgene expression is predominantly in microglia
Insert	Apoe apolipoprotein E [Mus musculus] (NM_009696.4)	Target gene
Restriction Sites	HindIII / XbaI	Precise cloning sites to ensure correct orientation and integrity of the insert
Primer Sequences	- Y20446-F: ccgtcagatcactagaagcttGCCACCatgaaggctctgtgggccgtgctgttggtcac<br>- Y20446-R: gcggccctagaattaTCTAGAtcattgattctcctgggccactggggtgatgatggggt	PCR amplification primers incorporating HindIII/XbaI sites at their 5′ ends
Plasmid Validation	Restriction digest + Sanger sequencing	Dual verification of correct construction and to exclude insert mutations or frame shifts
Plasmid Purity	Endotoxin-free (ultrapure) prep	High-quality plasmid is critical for efficient AAV packaging and transduction

Table S5. Statistical analysis values for the main figures.

Panel	Comparisons	Statistical test	Statistic (value, df)	P-value	Effect size
Fig 3C	TLE-NC vs TLE-HS	t-test	t(5)=–6.96	P=0.0009	Cohen's d =-2.84
Fig 3F (Left)	IBA1-CS vs IBA1-IS	t-test	t(5)=8.51	P=0.0002	Cohen's d=3.47
Fig 3F (Left)	APOE-CS vs APOE-IS	t-test	t(5)=20.42	P<0.0001	Cohen's d=8.34
Fig 3F (Right)	IBA1/APOE, GFAP/APOE, NeuN/APOE	One-way ANOVA	F(2, 18)=126.85	P<0.0001	partial η^2 = 0.93
Fig 3F (Right)	GFAP/APOE vs IBA1/APOE	Tukey's HSD	Mean diff=0.52	P<0.0001	Cohen's d =2.51
Fig 3F (Right)	GFAP/APOE vsNeuN/APOE	Tukey's HSD	Mean diff=–0.37	P=0.0002	Cohen's d=1.87
Fig 3F (Right)	IBA1/APOE vs NeuN/APOE	Tukey's HSD	Mean diff=–0.90	P<0.0001	Cohen's d=7.99
Fig 4E	Main effect of KA	Two-way ANOVA	F(1, 20)=121.91	P<0.0001	partial η^2=0.86
Fig 4E	Main effect of Time	Two-way ANOVA	F(1, 20)=33.38	P<0.0001	partial η^2=0.63
Fig 4E	KA × Time interaction	Two-way ANOVA	F(1, 20)=30.85	P<0.0001	partial η^2=0.61
Fig 4E	3 d NC vs 3 d KA	Tukey's HSD	mean diff=0.44	p=0.0036	Cohen's d=3.05
Fig 4E	14 d NC vs 14 d KA	Tukey's HSD	mean diff=1.33	P=0.0002	Cohen's d=5.61
Fig 4E	3 d NC vs 14 d NC	Tukey's HSD	mean diff=0.02	P=0.9985	Cohen's d=0.12
Fig 4E	3 d KA vs 14 d KA	Tukey's HSD	mean diff=0.91	P=0.0044	Cohen's d=3.96
Fig 5C-TNF α	Main effect of APOE	Two-way ANOVA	F(1, 8)=19.23	P=0.0023	partial η^2=0.71
Fig 5C-TNF α	Main effect of KA	Two-way ANOVA	F(1, 8)=378.92	P<0.0001	partial η^2=0.98

Fig 5C-TNF α	APOE × KA interaction	Two-way ANOVA	F(1, 8)=0.17	P=0.6950	partial η^2=0.02
Fig 5C-TNF α	NC+PBS vs NC+KA	Tukey HSD	mean diff=16.405	P=0.0051	Cohen’s d=11.62
Fig 5C-TNF α	OE+PBS vs OE+KA	Tukey HSD	mean diff=15.73	P=0.0072	Cohen’s d=10.87
Fig 5C-TNF α	NC+PBS vs OE+PBS	Tukey HSD	mean diff=3.96	P=0.0465	Cohen’s d=2.50
Fig 5C-TNF α	NC+KA vs OE+KA	Tukey HSD	mean diff=3.29	P=0.0166	Cohen’s d=2.61
Fig 5C-TNF α	Main effect of KA	Two-way ANOVA	F(1, 8)=507.64	P<0.0001	partial η^2=0.98
Fig 5C-TNF α	Main effect of Inhibitor	Two-way ANOVA	F(1, 8)=6.12	P=0.0385	partial η^2=0.43
Fig 5C-TNF α	KA × Inhibitor interaction	Two-way ANOVA	F(1, 8)=0.073	P=0.794	partial η^2=0.01
Fig 5C-TNF α	OE+PBS vs OE+PBS+Inh	Tukey HSD	mean diff=–1.52	P=0.4505	Cohen’s d=1.12
Fig 5C-TNF α	KA+PBS vs KA+PBS+Inh	Tukey HSD	mean diff=–1.89	P=0.0481	Cohen’s d=1.87
Fig 5C-IL 1β	Main effect of APOE	Two-way ANOVA	F(1, 8)=71.85	P<0.0001	partial η^2=0.90
Fig 5C-IL 1β	Main effect of KA	Two-way ANOVA	F(1, 8)=227.39	P<0.0001	partial η^2=0.97
Fig 5C-IL 1β	APOE × KA interaction	Two-way ANOVA	F(1, 8)=2.53	P=0.1504	partial η^2=0.24
Fig 5C-IL 1β	NC+PBS vs NC+KA	Tukey’s HSD	mean diff=22.14	P< 0.0001	Cohen’s d=12.49
Fig 5C-IL 1β	OE+PBS vs OE+KA	Tukey’s HSD	mean diff=17.92	P=0.0273	Cohen’s d=6.57
Fig 5C-IL 1β	NC+PBS vs OE+PBS	Tukey’s HSD	mean diff=13.37	P< 0.0001	Cohen’s d=6.25
Fig 5C-IL 1β	NC+KA vs OE+KA	Tukey’s HSD	mean diff=9.15	P=0.0012	Cohen’s d=3.73
Fig 5C-IL 1β	Main effect of KA	Two-way ANOVA	F(1, 8)=176.06	P< 0.0001	partial η^2=0.96

Fig 5C-IL 1β	Main effect of Inhibitor	Two-way ANOVA	F(1, 8)=33.82	P< 0.0001	partial η^2=0.81
Fig 5C-IL 1β	KA × Inhibitor interaction	Two-way ANOVA	F(1, 8)=2.42	P=0.1587	partial η^2=0.23
Fig 5C-IL 1β	OE+PBS vs OE+PBS+Inh	Tukey's HSD	mean diff=–5.15	P=0.0489	Cohen's d=3.59
Fig 5C-IL 1β	OE+KA vs OE+KA+Inh	Tukey's HSD	mean diff=–8.91	P=0.0015	Cohen's d=3.44
Fig 5D-IL6	Main effect of APOE	Two-way ANOVA	F(1, 8)=150.48	P< 0.0001	partial η^2=0.95
Fig 5D-IL6	Main effect of KA	Two-way ANOVA	F(1, 8)=191.25	P< 0.0001	partial η^2=0.96
Fig 5D-IL6	APOE × KA interaction	Two-way ANOVA	F(1, 8)=0.034	P=0.8589	partial η^2=0.001
Fig 5D-IL6	NC+PBS vs NC+KA	Tukey's HSD	mean diff=21.00	P=0.0035	Cohen's d=17.43
Fig 5D-IL6	OE+PBS vs OE+KA	Tukey's HSD	mean diff=20.45	P=0.0273	Cohen's d=5.90
Fig 5D-IL6	NC+PBS vs OE+PBS	Tukey's HSD	mean diff=18.66	P< 0.0001	Cohen's d=10.17
Fig 5D-IL6	NC+KA vs OE+KA	Tukey's HSD	mean diff=18.11	P=0.0199	Cohen's d=5.70
Fig 5D-IL6	Main effect of KA	Two-way ANOVA	F(1, 8)=157.43	P< 0.0001	partial η^2=0.95
Fig 5D-IL6	Main effect of Inhibitor	Two-way ANOVA	F(1, 8)=50.12	P< 0.0001	partial η^2=0.86
Fig 5D-IL6	KA × Inhibitor interaction	Two-way ANOVA	F(1, 8)=0.13	P=0.7233	partial η^2=0.02
Fig 5D-IL6	OE+PBS vs OE+PBS+Inh	Tukey's HSD	mean diff=–12.50	P=0.0041	Cohen's d=6.19
Fig 5D-IL6	OE+KA vs OE+KA+Inh	Tukey's HSD	mean diff=–11.27	P=0.0091	Cohen's d=3.15
Fig 5F–TLR4	Main effect of APOE	Two-way ANOVA	F(1, 8)=114.23	P< 0.0001	partial η^2=0.93

Fig 5F–TLR4	Main effect of KA	Two-way ANOVA	F(1, 8)=135.44	P< 0.0001	partial η²=0.94
Fig 5F–TLR4	APOE × KA interaction	Two-way ANOVA	F(1, 8)=20.56	P=0.0019	partial η²=0.72
Fig 5F–TLR4	NC+PBS vs NC+KA	Tukey's HSD	mean diff=0.49	P=0.0012	Cohen's d=6.86
Fig 5F–TLR4	OE+PBS vs OE+KA	Tukey's HSD	mean diff=1.11	P=0.0018	Cohen's d=7.29
Fig 5F–TLR4	NC+PBS vs OE+PBS	Tukey's HSD	mean diff=0.42	P=0.0094	Cohen's d=4.28
Fig 5F–TLR4	NC+KA vs OE+KA	Tukey's HSD	mean diff=1.04	P=0.0051	Cohen's d=7.67
Fig 5F–TLR4	Main effect of KA	Two-way ANOVA	F(1, 8)=131.82	P< 0.0001	partial η²=0.94
Fig 5F–TLR4	Main effect of Inhibitor	Two-way ANOVA	F(1, 8)=44.12	P< 0.0001	partial η²=0.85
Fig 5F–TLR4	KA × Inhibitor interaction	Two-way ANOVA	F(1, 8)=8.93	P=0.0174	partial η²=0.53
Fig 5F–TLR4	OE+PBS vs OE+PBS+Inh	Tukey's HSD	mean diff=–0.28	P=0.0328	Cohen's d=2.70
Fig 5F–TLR4	OE+KA vs OE+KA+Inh	Tukey's HSD	mean diff=–0.74	P=0.0062	Cohen's d=4.72
Fig 5F-cGAS	Main effect of APOE	Two-way ANOVA	F(1, 8)=56.95	P< 0.0001	partial η²=0.88
Fig 5F-cGAS	Main effect of KA	Two-way ANOVA	F(1, 8)=72.68	P< 0.0001	partial η²=0.91
Fig 5F-cGAS	APOE × KA interaction	Two-way ANOVA	F(1, 8)=0.38	P=0.555	partial η²=0.05
Fig 5F-cGAS	NC+PBS vs NC+KA	Tukey's HSD	mean diff=0.97	P=0.0087	Cohen's d=5.16
Fig 5F-cGAS	OE+PBS vs OE+KA	Tukey's HSD	mean diff=1.120	P=0.0079	Cohen's d=4.78
Fig 5F-cGAS	NC+PBS vs OE+PBS	Tukey's HSD	mean diff=0.85	P=0.0025	Cohen's d=3.58
Fig 5F-cGAS	NC+KA vs OE+KA	Tukey's HSD	mean diff=1.00	P=0.0201	Cohen's d=5.45

Fig 5F-cGAS	Main effect of KA	Two-way ANOVA	F(1, 8)=79.20	P< 0.0001	partial η²=0.91
Fig 5F-cGAS	Main effect of Inhibitor	Two-way ANOVA	F(1, 8)=12.66	P=0.0074	partial η²=0.61
Fig 5F-cGAS	KA × Inhibitor interaction	Two-way ANOVA	F(1, 8)=0.71	P=0.4231	partial η²=0.08
Fig 5F-cGAS	OE+PBS vs OE+PBS+Inh	Tukey's HSD	mean diff=–0.31	P=0.0415	Cohen's d=1.34
Fig 5F-cGAS	OE+KA vs OE+KA+Inh	Tukey's HSD	mean diff=–0.51	P=0.0432	Cohen's d=3.21
Fig 5F-pSTING	Main effect of APOE	Two-way ANOVA	F(1, 8)=210.11	P< 0.0001	partial η² = 0.96
Fig 5F-pSTING	Main effect of KA	Two-way ANOVA	F(1, 8)=521.83	P< 0.0001	partial η² = 0.99
Fig 5F-pSTING	APOE × KA interaction	Two-way ANOVA	F(1, 8)=0.46	P=0.5141	partial η² = 0.05
Fig 5F-pSTING	NC+PBS vs NC+KA	Tukey's HSD	mean diff=1.78	P=0.0040	Cohen's d = 7.78
Fig 5F-pSTING	OE+PBS vs OE+KA	Tukey's HSD	mean diff=1.83	P=0.0299	Cohen's d = 3.63
Fig 5F-pSTING	NC+PBS vs OE+PBS	Tukey's HSD	mean diff=1.51	P=0.0044	Cohen's d = 4.78
Fig 5F-pSTING	NC+KA vs OE+KA	Tukey's HSD	mean diff=1.56	P=0.0079	Cohen's d = 3.43
Fig 5F-pSTING	Main effect of KA	Two-way ANOVA	F(1, 8)=97.16	P< 0.0001	partial η² = 0.92
Fig 5F-pSTING	Main effect of Inhibitor	Two-way ANOVA	F(1, 8)=28.97	P< 0.0001	partial η² = 0.78
Fig 5F-pSTING	KA × Inhibitor interaction	Two-way ANOVA	F(1, 8)=1.34	P=0.2803	partial η² = 0.14
Fig 5F-pSTING	OE+PBS vs OE+PBS+Inh	Tukey's HSD	mean diff=–1.38	P=0.0358	Cohen's d = 5.52
Fig 5F-pSTING	OE+KA vs OE+KA+Inh	Tukey's HSD	mean diff=–0.89	P=0.0063	Cohen's d = 1.97

Fig 5F-STING	Main effect of APOE	Two-way ANOVA	$F(1, 8)=28.08$	$P< 0.0001$	partial $\eta^2=0.78$
Fig 5F-STING	Main effect of KA	Two-way ANOVA	$F(1, 8)=51.05$	$P< 0.0001$	partial $\eta^2=0.87$
Fig 5F-STING	APOE × KA interaction	Two-way ANOVA	$F(1, 8)=1.37$	$P=0.2766$	partial $\eta^2=0.15$
Fig 5F-STING	NC+PBS vs NC+KA	Tukey's HSD	mean diff=0.98	$P=0.0451$	Cohen's d=7.33
Fig 5F-STING	OE+PBS vs OE+KA	Tukey's HSD	mean diff=0.71	$P=0.0028$	Cohen's d=2.75
Fig 5F-STING	NC+PBS vs OE+PBS	Tukey's HSD	mean diff=0.76	$P=0.0309$	Cohen's d=4.02
Fig 5F-STING	NC+KA vs OE+KA	Tukey's HSD	mean diff=0.49	$P=0.0365$	Cohen's d=2.23
Fig 5F-STING	Main effect of KA	Two-way ANOVA	$F(1, 8)=10.52$	$P=0.0118$	partial $\eta^2=0.57$
Fig 5F-STING	Main effect of Inhibitor	Two-way ANOVA	$F(1, 8)=4.73$	$P=0.0614$	partial $\eta^2=0.37$
Fig 5F-STING	KA × Inhibitor interaction	Two-way ANOVA	$F(1, 8)=8.09$	$P=0.0217$	partial $\eta^2=0.50$
Fig 5F-STING	OE+PBS vs OE+PBS+Inh	Tukey's HSD	mean diff=0.08	$P=0.9628$	Cohen's d=0.42
Fig 5F-STING	OE+KA vs OE+KA+Inh	Tukey's HSD	mean diff=–0.58	$P=0.0358$	Cohen's d=2.70
Fig 5F-APOE	Main effect of APOE	Two-way ANOVA	$F(1, 8)=64.01$	$P< 0.0001$	partial $\eta^2=0.89$
Fig 5F-APOE	Main effect of KA	Two-way ANOVA	$F(1, 8)=13.07$	$P=0.0068$	partial $\eta^2=0.62$
Fig 5F-APOE	APOE × KA interaction	Two-way ANOVA	$F(1, 8)=0.19$	$P=0.6741$	partial $\eta^2=0.02$
Fig 5F-APOE	NC+PBS vs NC+KA	Tukey's HSD	mean diff=0.31	$P=0.0367$	Cohen's d=1.99
Fig 5F-APOE	OE+PBS vs OE+KA	Tukey's HSD	mean diff=0.39	$P=0.0298$	Cohen's d=2.18

Fig 5F-APOE	NC+PBS vs OE+PBS	Tukey's HSD	mean diff=0.74	P< 0.0001	Cohen's d=5.08
Fig 5F-APOE	NC+KA vs OE+KA	Tukey's HSD	mean diff=1.14	P=0.0014	Cohen's d=4.34
Fig 5F-APOE	Main effect of KA	Two-way ANOVA	F(1, 8)=32.97	P< 0.0001	partial η^2=0.81
Fig 5F-APOE	Main effect of Inhibitor	Two-way ANOVA	F(1, 8)=0.43	P=0.5299	partial η^2=0.05
Fig 5F-APOE	KA × Inhibitor interaction	Two-way ANOVA	F(1, 8)=1.11	P=0.3236	partial η^2=0.12
Fig 5F-APOE	OE+PBS vs OE+PBS+Inh	Tukey's HSD	mean diff = –0.14	P=0.6392	Cohen's d=1.33
Fig 5F-APOE	OE+KA vs OE+KA+Inh	Tukey's HSD	mean diff = 0.034	P=0.9918	Cohen's d=0.19
Fig 6D-IBA1	Main effect of genotype	Two-way ANOVA	F(1, 20)=307.02	P< 0.0001	partial η^2 = 0.94
Fig 6D-IBA1	Main effect of side	Two-way ANOVA	F(1, 20)=118.06	P< 0.0001	partial η^2 = 0.86
Fig 6D-IBA1	Genotype × Side interaction	Two-way ANOVA	F(1, 20)=115.15	P< 0.0001	partial η^2 = 0.85
Fig 6D-IBA1	WT-CS vs WT-IS	Tukey's HSD	mean diff=1.55	P< 0.0001	Cohen's d = 6.38
Fig 6D-IBA1	KO-CS vs KO-IS	Tukey's HSD	mean diff=0.01	P=0.9997	Cohen's d = 0.18
Fig 6D-IBA1	WT-CS vs KO-CS	Tukey's HSD	mean diff=0.49	P=0.0004	Cohen's d = 2.99
Fig 6D-IBA1	WT-IS vs KO-IS	Tukey's HSD	mean diff=2.03	P< 0.0001	Cohen's d = 10.81
Fig 6D-GFAP	Main effect of genotype	Two-way ANOVA	F(1, 20)=7.27	P=0.0139	partial η^2=0.27
Fig 6D-GFAP	Main effect of side	Two-way ANOVA	F(1, 20)=36.06	P< 0.0001	partial η^2=0.64
Fig 6D-GFAP	Genotype × Side interaction	Two-way ANOVA	F(1, 20)=20.58	P< 0.0001	partial η^2=0.51

Fig 6D-GFAP	WT-CS vs WT-IS	Tukey's HSD	mean diff=0.56	$P< 0.0001$	Cohen's d=3.92
Fig 6D-GFAP	KO-CS vs KO-IS	Tukey's HSD	mean diff=0.08	$P=0.7295$	Cohen's d=0.67
Fig 6D-GFAP	WT-CS vs KO-CS	Tukey's HSD	mean diff=0.10	$P=0.5723$	Cohen's d=1.24
Fig 6D-GFAP	WT-IS vs KO-IS	Tukey's HSD	mean diff=0.38	$P=0.0477$	Cohen's d=2.31
Fig 6D-NeuN	Main effect of genotype	Two-way ANOVA	$F(1, 20)=57.87$	$P< 0.0001$	partial $\eta^2 = 0.74$
Fig 6D-NeuN	Main effect of side	Two-way ANOVA	$F(1, 20)=24.74$	$P< 0.0001$	partial $\eta^2 = 0.55$
Fig 6D-NeuN	Genotype × Side interaction	Two-way ANOVA	$F(1, 20)=4.94$	$P=0.0379$	partial $\eta^2 = 0.19$
Fig 6D-NeuN	WT-CS vs WT-IS	Tukey's HSD	mean diff=–0.41	$P=0.0021$	Cohen's d = 2.37
Fig 6D-NeuN	KO-CS vs KO-IS	Tukey's HSD	mean diff=–0.16	$P=0.2418$	Cohen's d = 1.65
Fig 6D-NeuN	WT-CS vs KO-CS	Tukey's HSD	mean diff=0.31	$P=0.0126$	Cohen's d = 1.75
Fig 6D-NeuN	WT-IS vs KO-IS	Tukey's HSD	mean diff=0.56	$P< 0.0001$	Cohen's d = 6.15
Fig 7F (Left)	Main effect of genotype	Two-way ANOVA	$F(1, 36)=4.39$	$P=0.0433$	partial η^2=0.11
Fig 7F (Left)	Main effect of AAV	Two-way ANOVA	$F(1, 36)=8.23$	$P=0.0069$	partial η^2=0.19
Fig 7F (Left)	Genotype × AAV interaction	Two-way ANOVA	$F(1, 36)=3.10$	$P=0.0869$	partial η^2=0.08
Fig 7F (Left)	$APOE^{-/-}$+AAV-Control+KA APOE-/-+AAV-APOE+KA	Tukey's HSD	Mean diff=3.83	$P=0.0121$	Cohen's d=1.69
Fig 7F (Left)	$APOE^{-/-}$+AAV-Control+KA WT+AAV-Control+KA	Tukey's HSD	Mean diff=3.19	$P=0.0464$	Cohen's d=1.24
Fig 7F (Left)	$APOE^{-/-}$+AAV-APOE+KA WT+AAV-APOE+KA	Tukey's HSD	Mean diff=0.28	$P=0.9952$	Cohen's d=0.10
Fig 7F (Left)	WT+AAV-Control+KA vs WT+AAV-APOE+KA	Tukey's HSD	Mean diff=0.92	$P=0.8614$	Cohen's d=0.31
Fig 7F (Left)	WT+AAV-Control vs	Unpaired t-test	Mean diff=3.45	$P=0.0161$	Cohen's d=1.19

	WT+AAV-Control+KA				
Fig 7F (Right)	Main effect of genotype	Two-way ANOVA	F(1,36)=2.52	P=0.1215	partial η^2=0.0653
Fig 7F (Right)	Main effect of AAV	Two-way ANOVA	F(1,36)=2.11	P=0.1549	partial η^2=0.0554
Fig 7F (Right)	Genotype × AAV interaction	Two-way ANOVA	F(1,36)=0.36	P=0.5506	partial η^2=0.0100
Fig 7F (Right)	$APOE^{-/-}$+AAV-Control+KA $APOE^{-/-}$+AAV-APOE+KA	Tukey's HSD	Mean diff=0.42	P=0.9310	Cohen's d=0.35
Fig 7F (Right)	$APOE^{-/-}$+AAV-Control+KA WT+AAV-Control+KA	Tukey's HSD	Mean diff=0.48	P=0.8981	Cohen's d=0.40
Fig 7F (Right)	$APOE^{-/-}$+AAV-APOE+KA WT+AAV-APOE+KA	Tukey's HSD	Mean diff=1.07	P=0.0420	Cohen's d=0.59
Fig 7F (Right)	WT+AAV-Control+KA WT+AAV-APOE+KA	Tukey's HSD	Mean diff=1.00	P=0.4755	Cohen's d=0.55
Fig 7F (Right)	WT+AAV-Control WT+AAV-Control+KA	t-test	Mean diff=1.28	P=0.0162	Cohen's d=1.25
Fig 7G (Left)	Main effect of genotype	Two-way ANOVA	F(1, 20)=125.07	P<0.0001	partial η^2= 0.86
Fig 7G (Left)	Main effect of AAV	Two-way ANOVA	F(1, 20)=115.52	P<0.0001	partial η^2= 0.85
Fig 7G (Left)	Genotype × AAV interaction	Two-way ANOVA	F(1, 20)=54.47	P<0.0001	partial η^2= 0.73
Fig 7G (Left)	$APOE^{-/-}$+AAV-Control+KA $APOE^{-/-}$+AAV-APOE+KA	Tukey's HSD	mean diff=1089.88	P<0.0001	Cohen's d = 5.67
Fig 7G (Left)	$APOE^{-/-}$+AAV-Control+KA WT+AAV-Control+KA	Tukey's HSD	mean diff=1116.08	P<0.0001	Cohen's d = 7.92
Fig 7G (Left)	$APOE^{-/-}$+AAV-APOE+KA WT+AAV-APOE+KA	Tukey's HSD	mean diff=228.67	P=0.0624	Cohen's d = 1.49
Fig 7G (Left)	WT+AAV-Control+KA	Tukey's HSD	mean diff=202.47	P=0.1133	Cohen's d = 2.54

	WT+AAV-APOE+KA				
Fig 7G (Middle)	Main effect of genotype	Two-way ANOVA	F(1, 20)=23.12	P=0.0001	partial η^2 = 0.54
Fig 7G (Middle)	Main effect of AAV	Two-way ANOVA	F(1, 20)=31.72	P<0.0001	partial η^2 = 0.61
Fig 7G (Middle)	Genotype × AAV interaction	Two-way ANOVA	F(1, 20)=6.02	P=0.0235	partial η^2 = 0.23
Fig 7G (Middle)	APOE$^{-/-}$+AAV-Control+KA APOE$^{-/-}$+AAV-APOE+KA	Tukey's HSD	mean diff=3.43	P=0.0001	Cohen's d = 4.03
Fig 7G (Middle)	APOE$^{-/-}$+AAV-Control+KA WT+AAV-Control+KA	Tukey's HSD	mean diff=3.08	P=0.0003	Cohen's d = 2.69
Fig 7G (Middle)	APOE$^{-/-}$+AAV-APOE+KA WT+AAV-APOE+KA	Tukey's HSD	mean diff=1.00	P=0.3671	Cohen's d = 1.08
Fig 7G (Middle)	WT+AAV-Control+KA WT+AAV-APOE+KA	Tukey's HSD	mean diff=1.35	P=0.0445	Cohen's d = 1.13
Fig 7G (Right)	Main effect of genotype	Two-way ANOVA	F(1, 20)=35.44	P<0.0001	partial η^2 = 0.64
Fig 7G (Right)	Main effect of AAV	Two-way ANOVA	F(1, 20)=21.53	P=0.0002	partial η^2 = 0.52
Fig 7G (Right)	Genotype × AAV interaction	Two-way ANOVA	F(1, 20)=0.00	P=0.9882	partial η^2 = 0.00
Fig 7G (Right)	APOE$^{-/-}$+AAV-Control+KA APOE$^{-/-}$+AAV-APOE+KA	Tukey's HSD	mean diff=26.00	P=0.0176	Cohen's d = -2.27
Fig 7G (Right)	APOE$^{-/-}$+AAV-Control+KA WT+AAV-Control+KA	Tukey's HSD	mean diff=33.33	P=0.0022	Cohen's d = -3.62
Fig 7G (Right)	APOE$^{-/-}$+AAV-APOE+KA WT+AAV-APOE+KA	Tukey's HSD	mean diff=33.17	P=0.0023	Cohen's d = -1.95

Fig 7G (Right)	WT+AAV-Control+KA<br>WT+AAV-APOE+KA	Tukey's HSD	mean diff=25.83	P=0.0184	Cohen's d = -1.66

Note: Table S5 summarizes all specific statistical test values presented in the main text and figures, including standardized effect sizes, complete test statistics, degrees of freedom, and p-values.

Figure S1. Base error rate distribution of the raw data (scRNA-seq quality control).

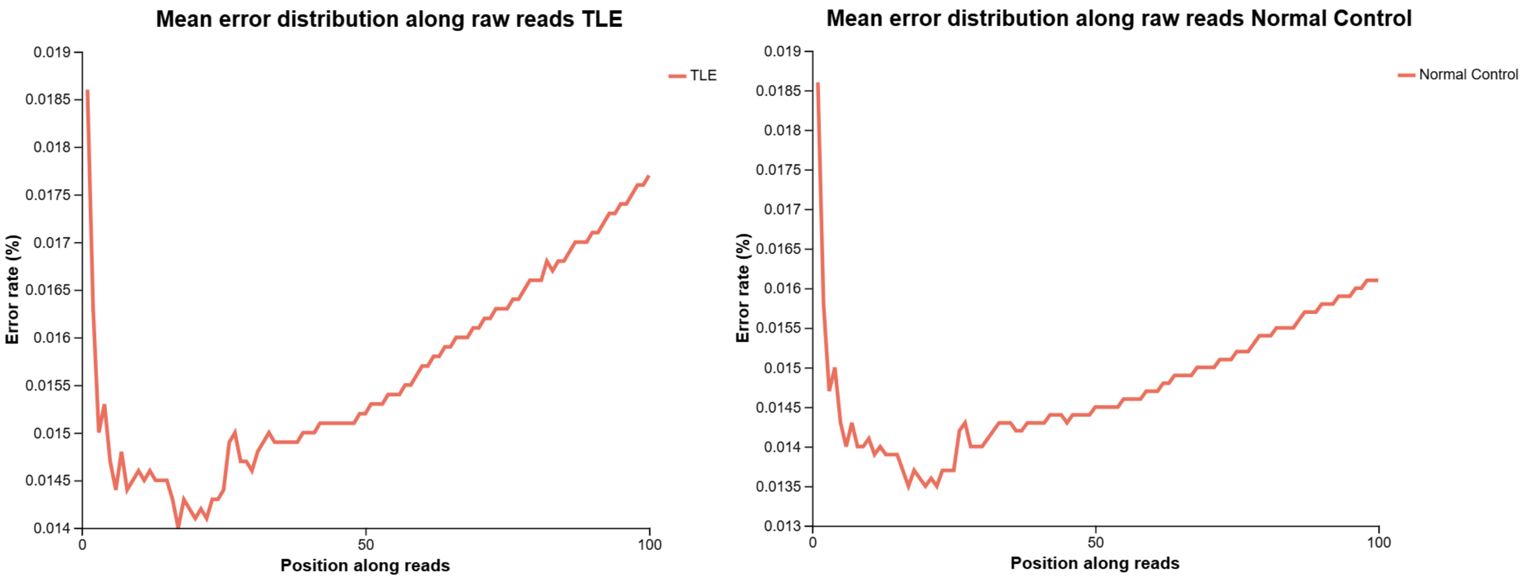

Figure S1 shows the mean base error rate for each base position of sequencing reads from the TLE (left) and control groups (right), respectively, which is generally below 0.02%. The horizontal axis represents the base position along the reads (from 5' to 3'), and the vertical axis indicates the average error rate at each position across all reads.

Figure S2. Base composition distribution plot (scRNA-seq quality control).

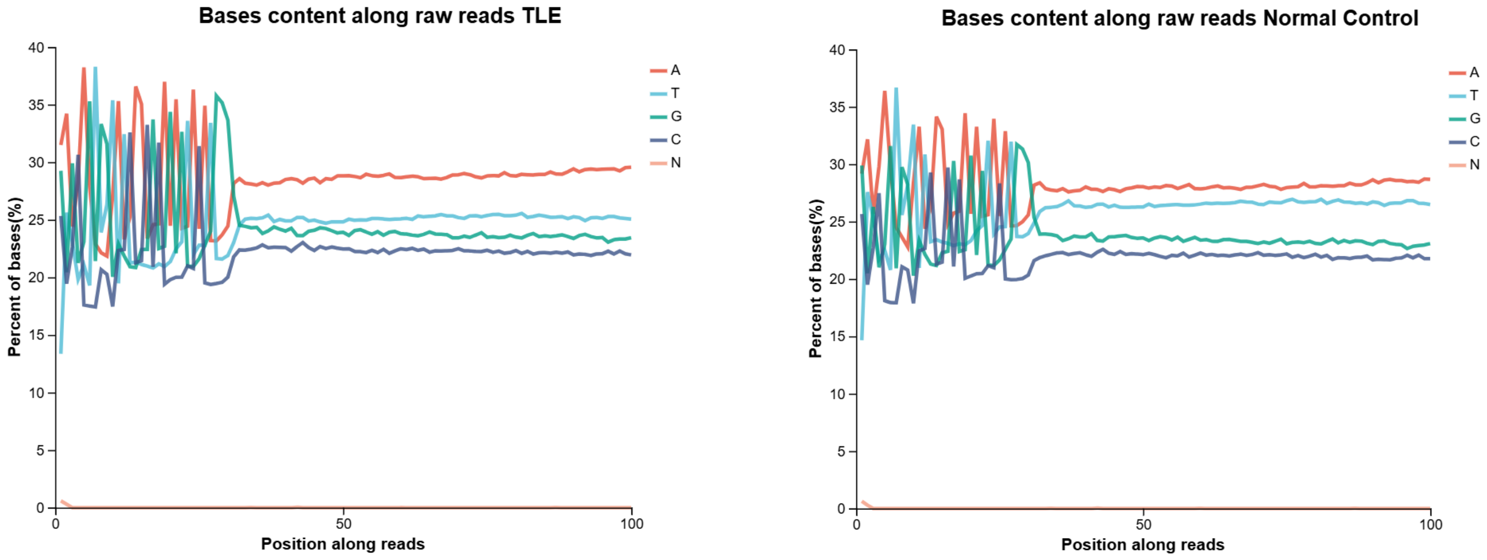

Figure S2 shows the mean bases content of sequencing reads from the TLE (left) and control groups (right), respectively. The horizontal axis represents base positions along each read, while the vertical axis shows the percentage of each base (A, C, G, T, and N) at that position across all reads (e.g., the first base of each read). Here, "N" denotes ambiguous bases that cannot be confidently identified as A, T, G, or C. Different colors indicate the various bases.

Figure S3. Bubble plot of marker gene expression.

Average expression and detection frequency of selected marker genes across the annotated cell types are shown in Figure S3. The horizontal axis shows cell types, and the vertical axis shows marker genes. (Cutt off value: LogFC=0.25, min.pct=0.25, adjusted P value <0.05).

After annotating cell types for each cluster, it displays the average expression levels of key marker genes across 12 cell types (astrocytes, choroid plexus cells, endothelial cells, erythrocytes, microglia, neural stem cells, neurons, oligodendrocytes, oligodendrocyte precursor cells, pericytes, T cells, and vascular leptomeningeal cells). Canonical marker genes reliably delineate each annotated cell type.

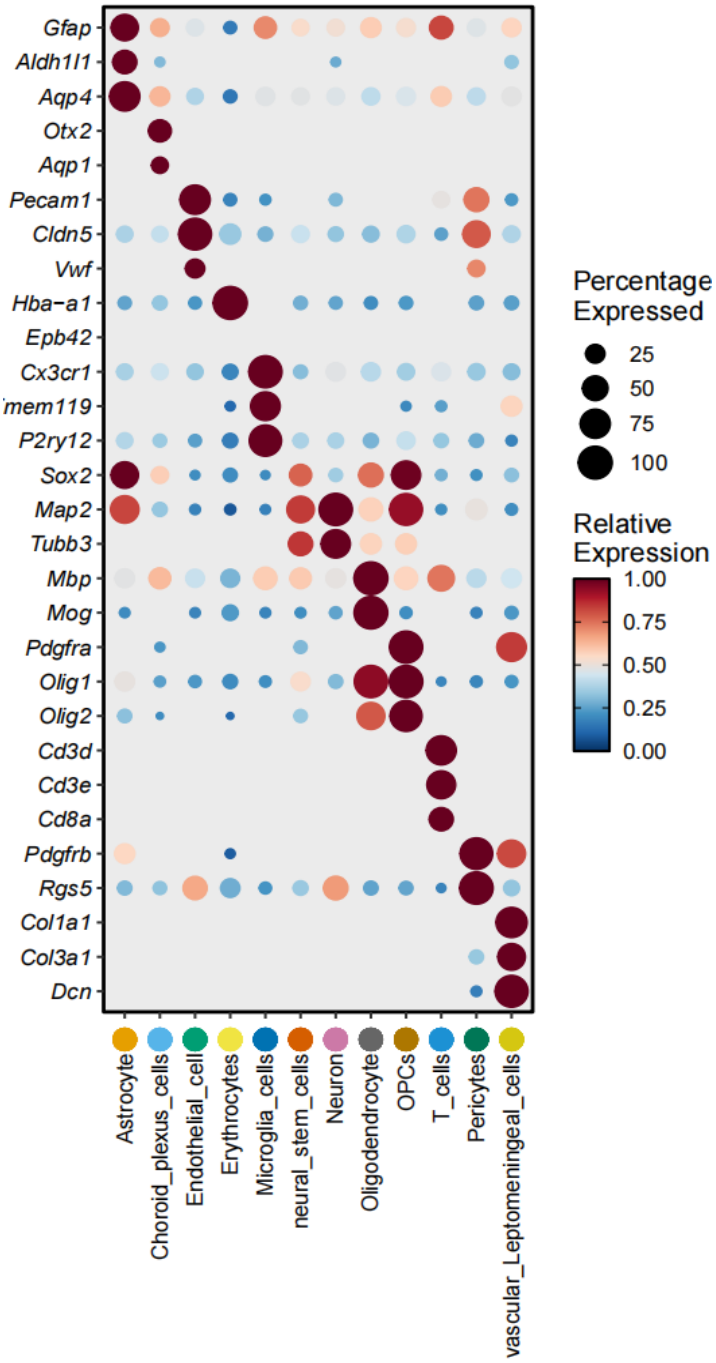

Figure S4. Expression of inflammatory cytokines in neuron-conditioned medium.

A

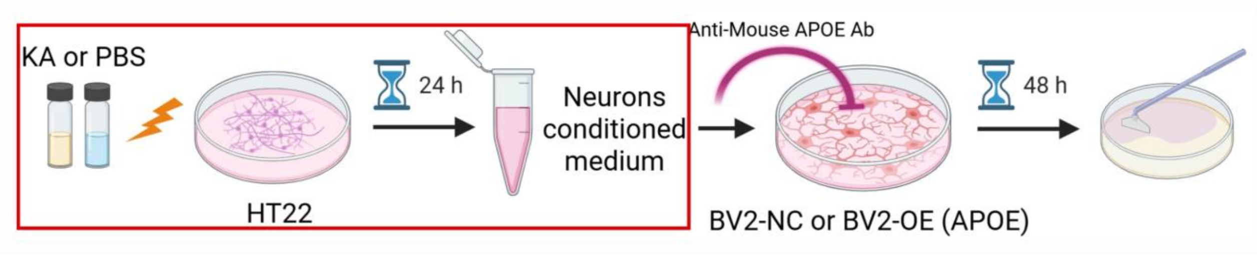

B

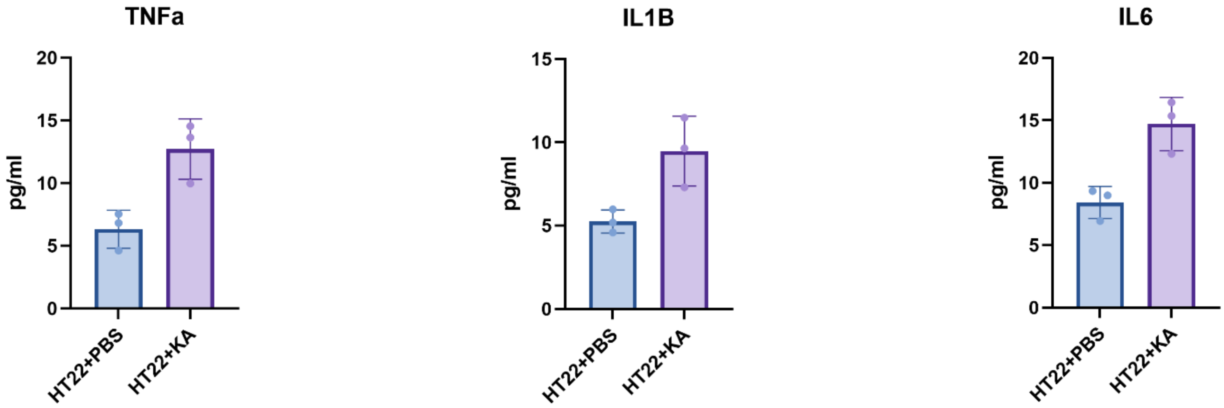

Figure S4 shows the expression of inflammatory cytokines in the supernatant of the co-culture experiment (HT22 hippocampal neurons treated with PBS or KA for 24 h). Figure S4A shows the overall workflow, consistent with main-text Figure 4B, focusing on levels of relevant markers in HT22 neuron–conditioned medium following 24 h pretreatment with KA or PBS. Figure S4B presents the levels of the cytokines TNFα, IL-1β and IL-6 in the conditioned medium for the experimental segment indicated by the red box in Figure S4A. Although KA stimulation induced an increase in inflammatory cytokine secretion by HT22 neurons, the overall levels remained very low.

Figure S5. Establishment of the TLE mouse and status epilepticus severity post-induction.

A

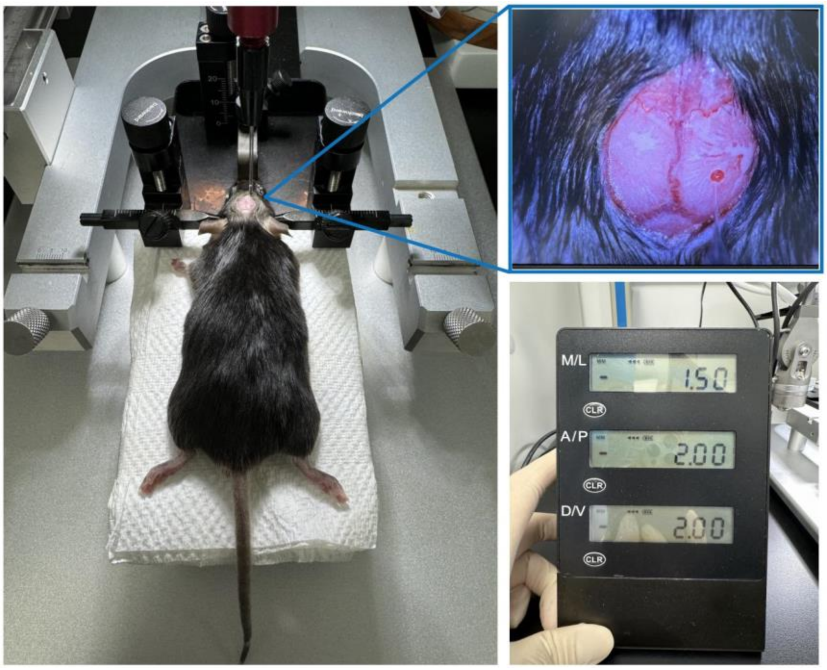

Figure S5A shows animal anesthesia and immobilization, followed by stereotaxic injection of kainic acid into the hippocampus to establish the TLE model. Details are provided in the Experimental Section/Methods.

B

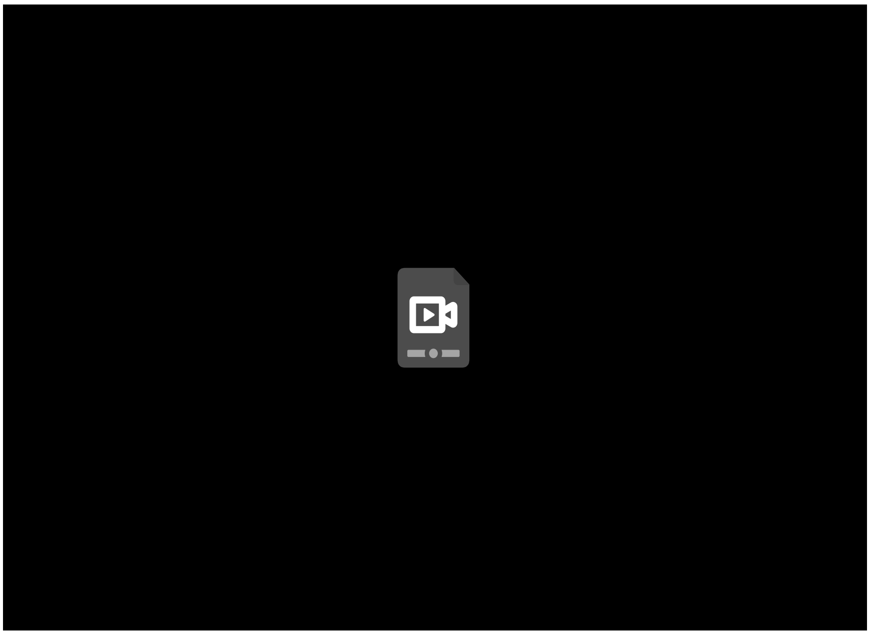

Figure S5B (Video). Real-time video recording of mouse behavior following stereotactic hippocampal injection of kainic acid in two hours. This clip captures a Racine stage 4-5 seizure, characterized by bilateral forelimb clonus with concurrent jerking of multiple limbs. Click the image to view the video.

C

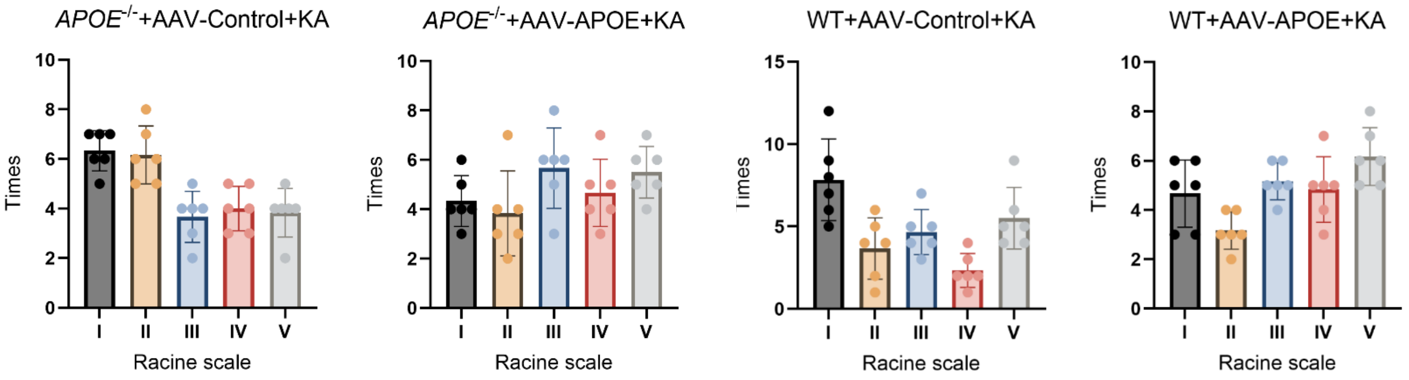

Figure S5C shows the status epilepticus severity based on the modified Racine scale (I. continuous ear and facial twitching with whisker trembling; II. head and neck jerks; III. forelimb clonus with rearing; IV. forelimb clonus accompanied by multi-limb twitching; V. generalized tonic–clonic seizures with loss of balance and falling down), recorded every five minutes for two hours after KA injection in the four experimental groups (*APOE*$^{-/-}$+AAV-Control+KA, *APOE*$^{-/-}$+AAV-APOE+KA, WT+AAV-Control+KA, and WT+AAV-*APOE*+KA).

Figure S6. Power spectral density (PSD) plot of four groups.

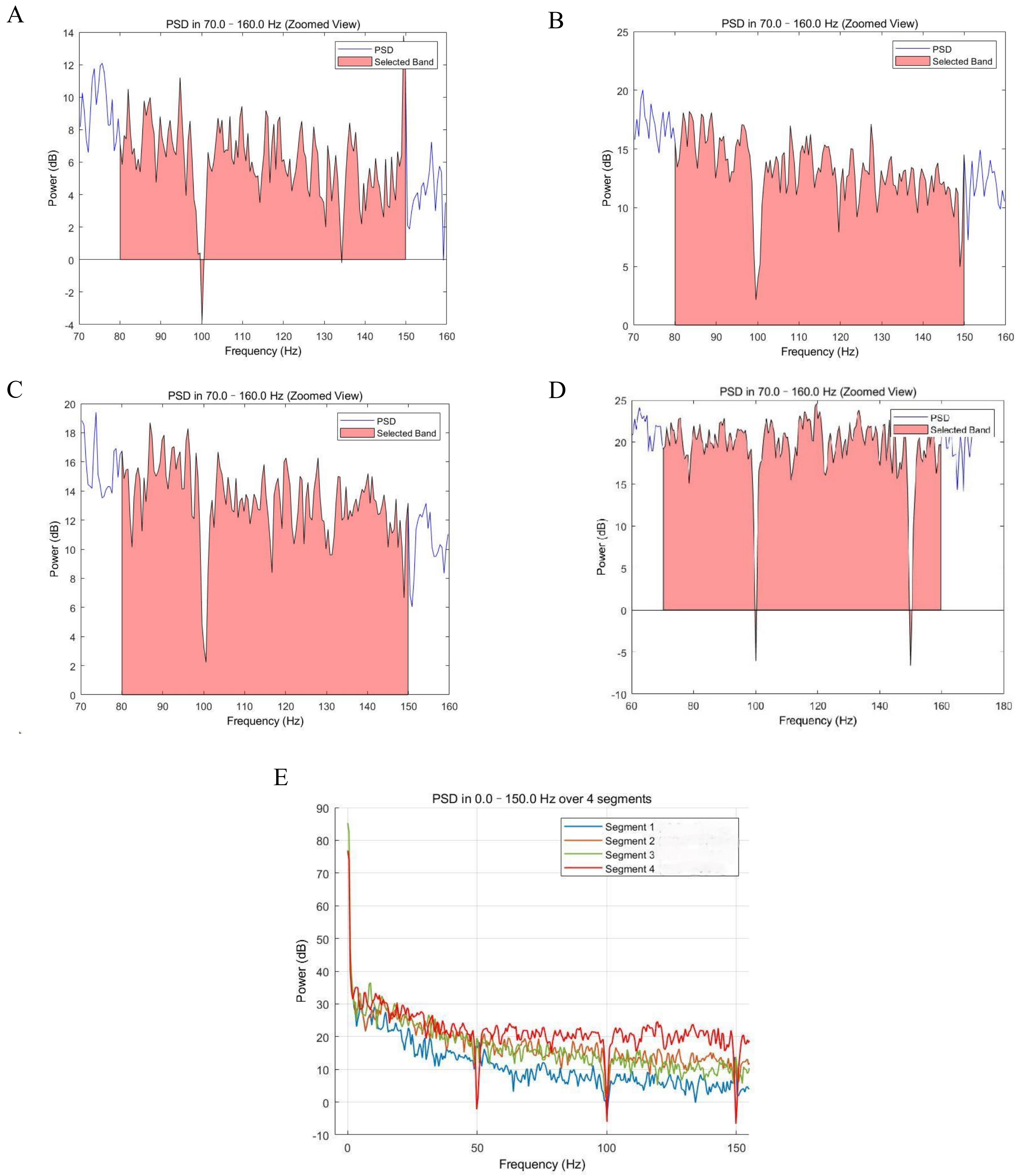

Figure S6 shows the fast Fourier transform–based power spectra of ictal EEG in the (A) *APOE*$^{-/-}$ + AAV-Control + KA group, (B) *APOE*$^{-/-}$ + AAV-*APOE* + KA group, (C) WT + AAV-Control + KA group, and (D) WT + AAV-*APOE* + KA group. Figure S6E shows the comparison of EEG PSD (0–150 Hz) across four groups. Caption PSD (dB) is shown for Group 1 (*APOE*$^{-/-}$ + AAV-Control + KA, blue), Group 2 (*APOE*$^{-/-}$ + AAV-*APOE* + KA, orange), Group 3 (WT + AAV-Control + KA, green) and Group 4 (WT + AAV-*APOE* + KA, red). A clear rise in high-frequency (80–150 Hz) activity can be observed in group 2, 3, and 4.

Figure S7. Video-EEG recordings from TLE mice and quantification of chronic-phase seizure severity using the modified Racine scale.

A

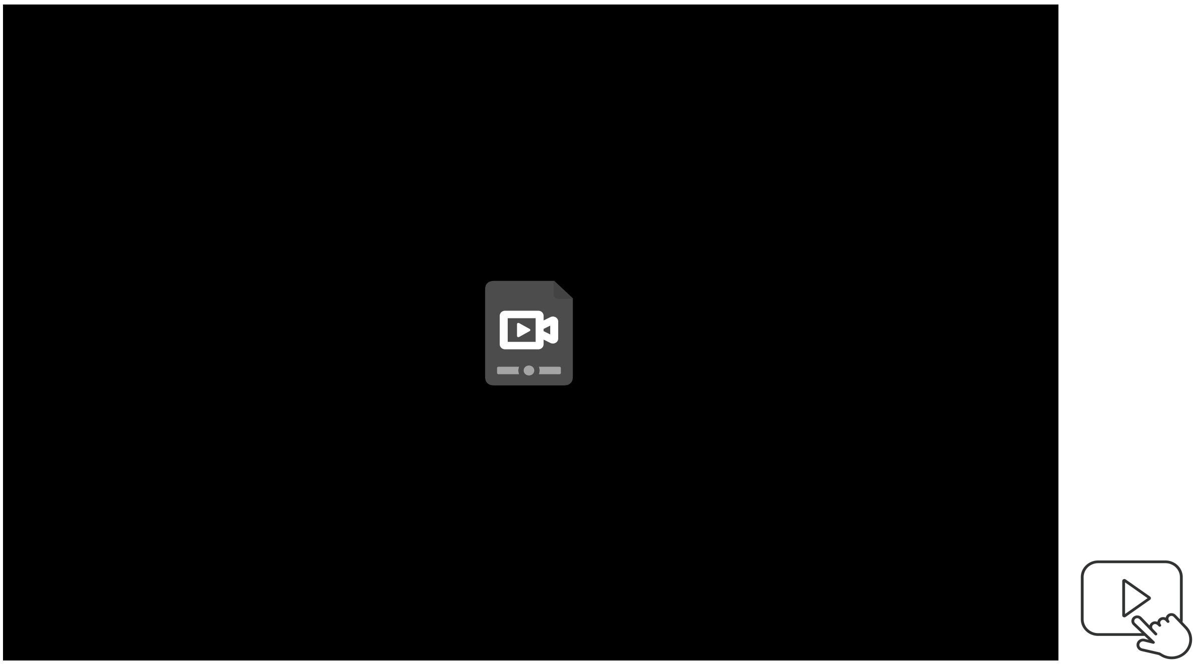

Figure S7A (Video). Combined video and EEG recordings were employed to capture and characterize seizure activity in the TLE mouse model. Click the image to view the video.

B

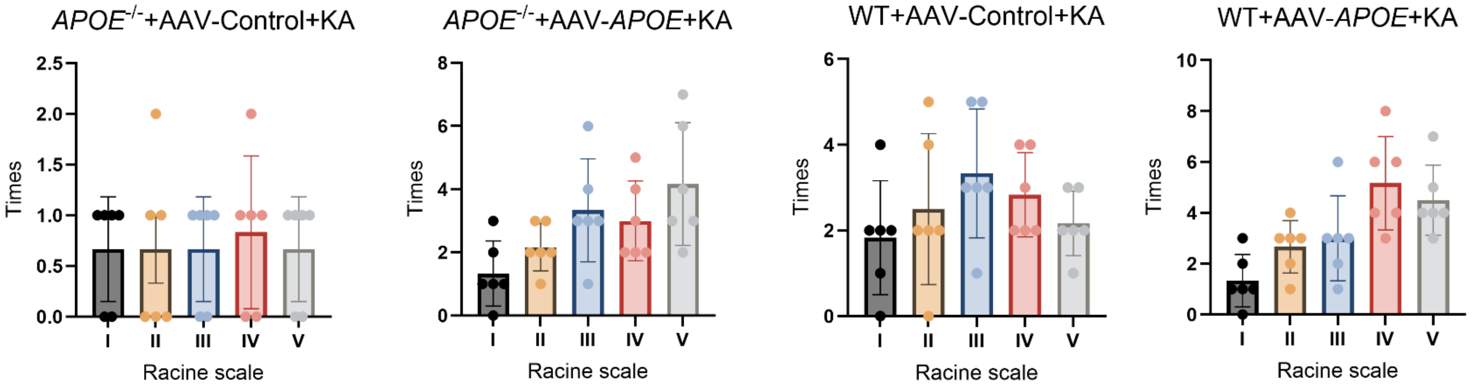

Figure S7B depicts four experimental cohorts (*APOE*$^{-/-}$+AAV-Control+KA, *APOE*$^{-/-}$+AAV-APOE+KA, WT+AAV-Control+KA, and WT+AAV-*APOE*+KA) undergoing continuous, 3-day seizure-severity monitoring beginning 14 days after KA injection, with assessments performed by synchronized video-EEG using the modified Racine scale (I. continuous ear and facial twitching with whisker trembling; II. head and neck jerks; III. forelimb clonus with rearing; IV. forelimb clonus accompanied by multi-limb twitching; V. generalized tonic–clonic seizures with loss of balance and falling down). Total seizure counts during the 3-day monitoring period were recorded and quantified. It corresponds to the middle panel of Figure 7G in the main text, which shows the mean number of seizures per day.

Figure S8. Metabolomics and lipidomics quality control.

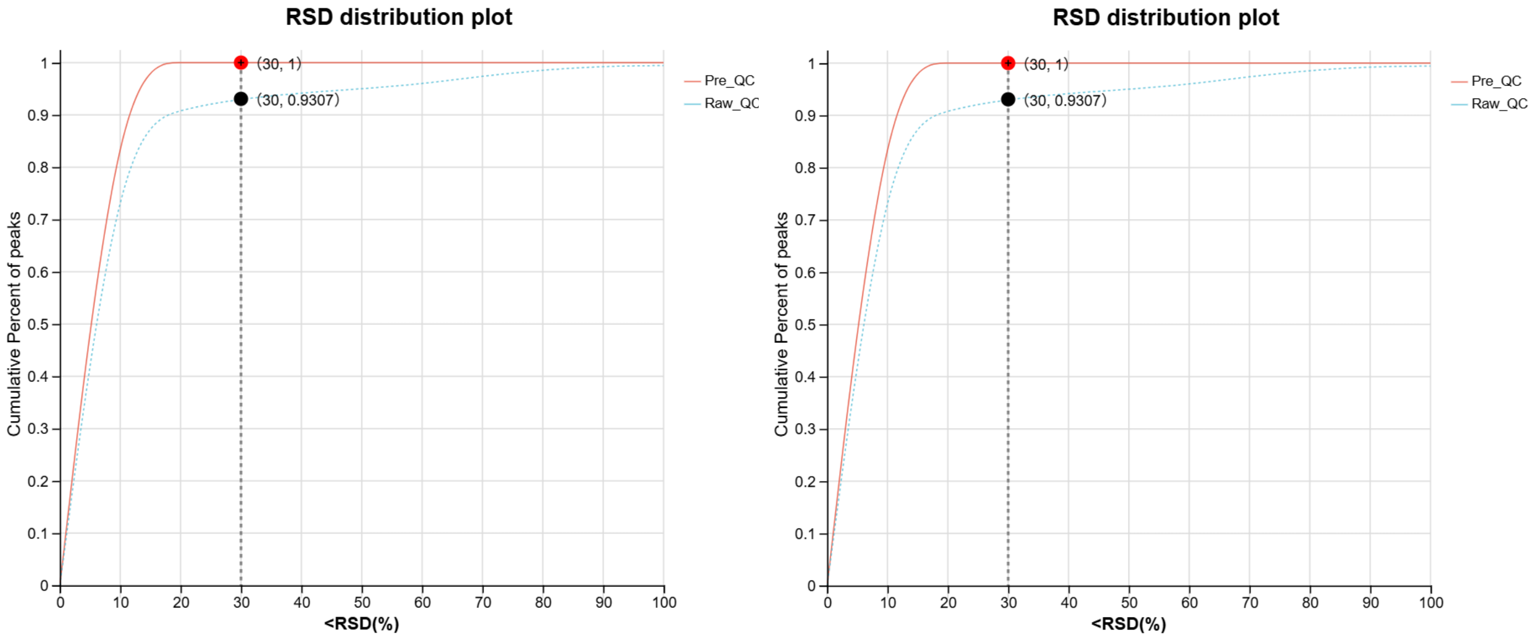

Raw metabolomics and lipidomics data were subjected to missing-value filtering, imputation, normalization, QC validation and transformation. The x-axis shows RSD (%) (standard deviation/mean) and the y-axis the cumulative proportion of ion peaks. Data are considered acceptable if RSD < 30% and the cumulative proportion > 0.7. Dashed lines indicate pre-processing and solid lines post-processing; raw data are represented by a single solid line. Left panel: full-scan LC; right panel: full-scan lipidomics.

Figure S9. Histopathological sections of hippocampal tissue in TLE patients.

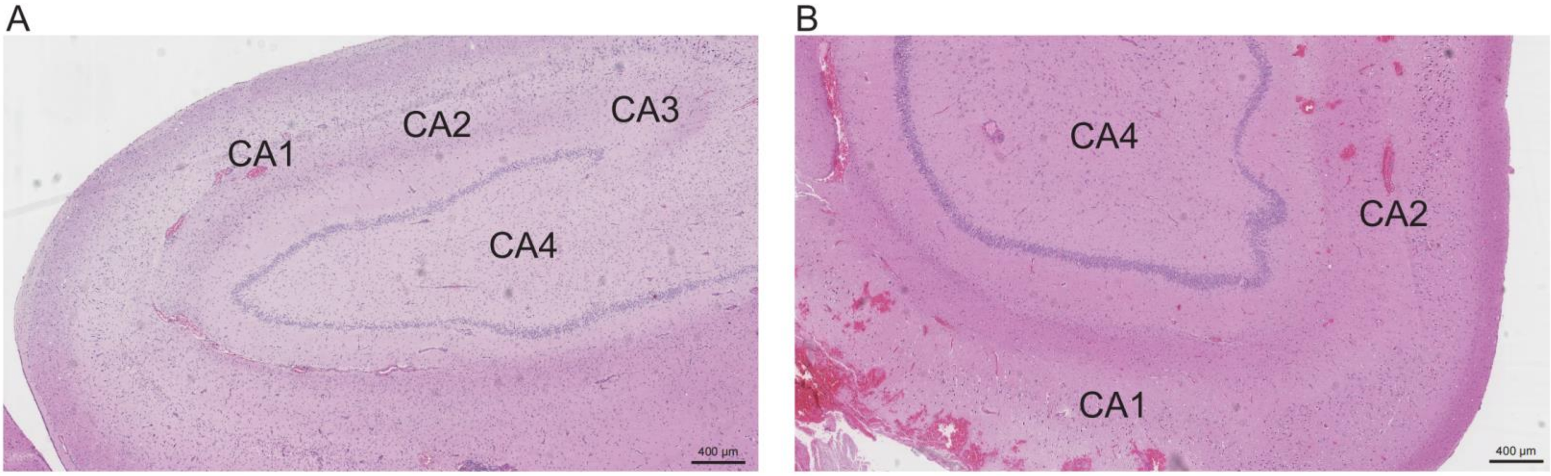

A. H&E Staining of TLE with HS. Gross Examination: A fixed brain tissue specimen from the right hippocampus, gray-white in color, slightly firm in texture, measuring approximately 1.9 × 0.6 × 1 cm. The tissue was sectioned coronally, perpendicular to the long axis of the hippocampus, revealing partial hippocampal structures. Pathological Diagnosis: Microscopic examination shows neuronal loss in the CA1 and CA4 regions, consistent with hippocampal sclerosis. Immunohistochemical Findings: GFAP (+), Olig-2(+), NeuN (+), Synaptophysin (+), Neurofilament (+), Calretinin (+). (scale bar, 400 μm)

B. H&E Staining of TLE without HS. Gross Examination: A fixed brain tissue specimen from the right hippocampus, gray-white in color, slightly firm in texture, measuring approximately 3.7 × 2 × 1.1 cm. The tissue was sectioned coronally, perpendicular to the long axis of the hippocampus, revealing partial hippocampal structures. Pathological Diagnosis: No significant neuronal loss observed in specific regions; however, focal gliosis is present. Immunohistochemical Findings: GFAP (+), Olig-2(+), NeuN (+), Synaptophysin (+), Neurofilament (+), Calretinin (+). (scale bar, 400 μm)

Figure S10. Cell state, morphology and EGFP reporter expression of BV2#mApoe and BV2#control (with qPCR validation)

A. Representative phase-contrast and fluorescence images of stable BV2 pools.

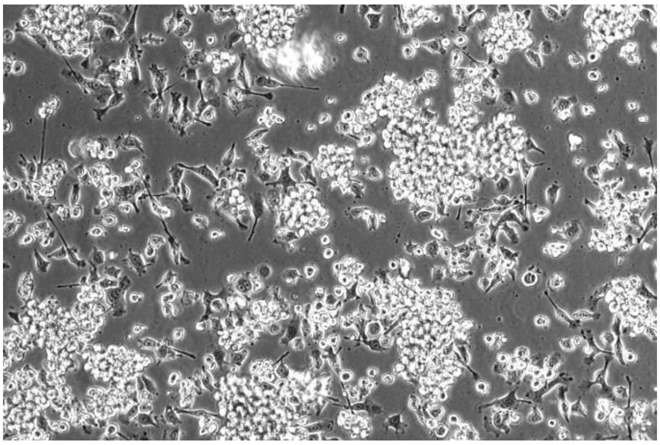

BV2#mApoe#Poolcells-100x-W

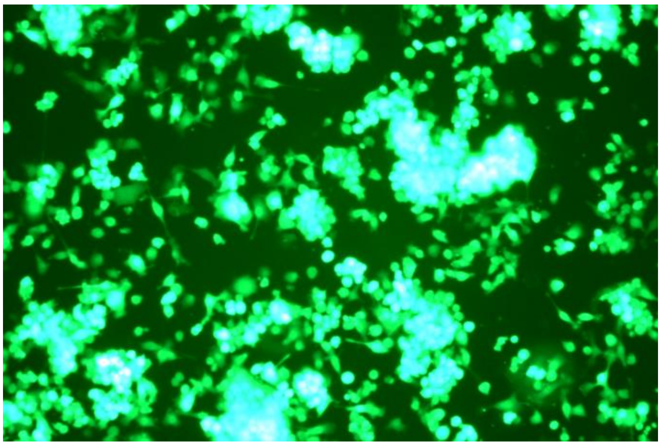

BV2#mApoe#Poolcells-100x-F(199MS)

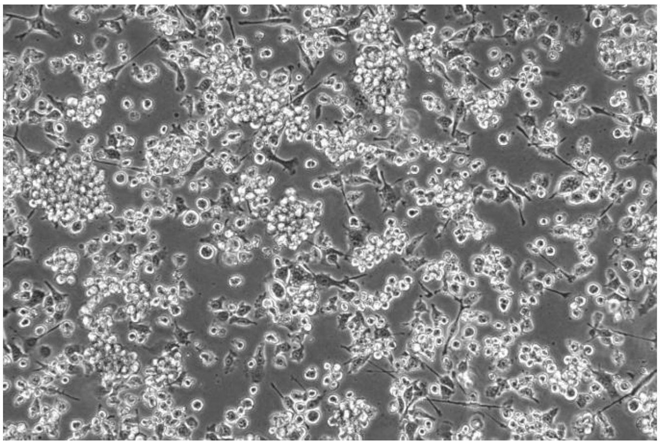

BV2#control#Poolcells-100x-W

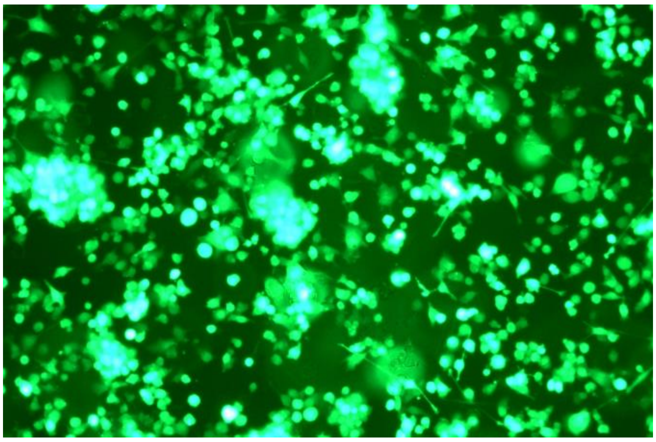

BV2#control#Poolcells-100x-F(199MS)

Left column: phase-contrast; right column: fluorescence (green = EGFP reporter indicating transgene expression). Top row: BV2#mApoe (pool; vector: pPB [Exp]-EF1A-EGFP>CAG-mApoe). Bottom row: BV2#control (pool; vector: pPB [Exp]-EF1A-EGFP>CAG-[ORF-stuffer]). Images were acquired with a 100× objective.

B. qPCR validation. (see Table S3 for primer information)

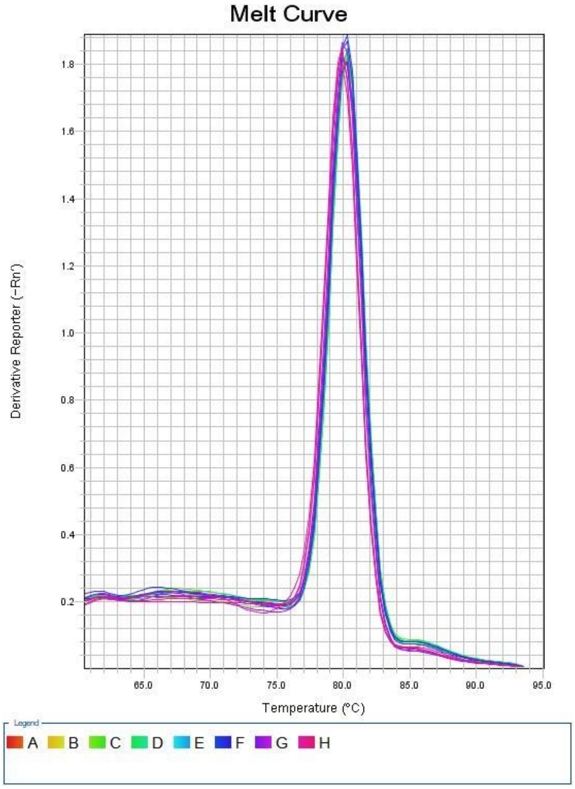

Melting curve for mApoe

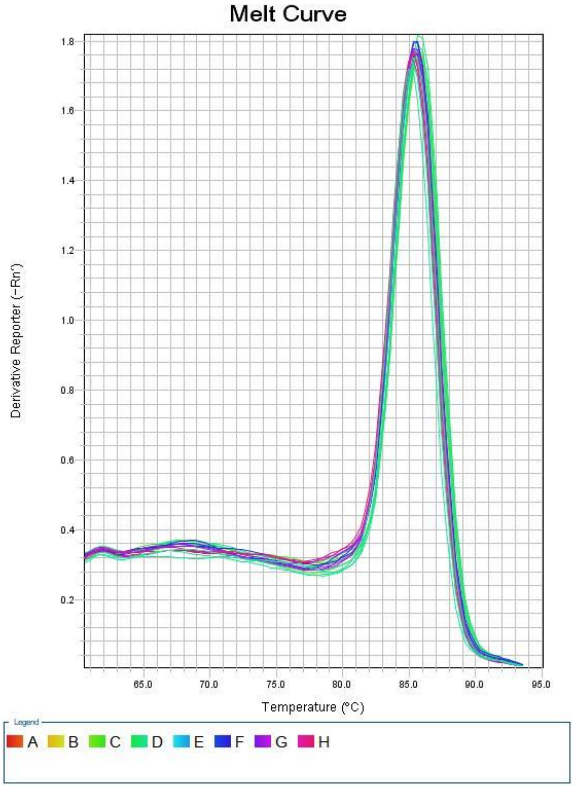

Melting curve for mGAPDH

Melting curves for mApoe (left) and mGAPDH (right), with qPCR indicating that mApoe expression in BV2#mApoe is 2.61-fold higher than in control.

Brief methods: BV2 stable pools (growth characteristic: semi-adherent / partial suspension) were generated by electroporation using the Neon™ system (Cat#MPK5000) and pooled after selection. Cells were maintained in DMEM-H supplemented with 10% FBS and 1% penicillin/streptomycin at 37°C in a humidified atmosphere of 95% air/5% CO_2. Cells were passaged at a split ratio of 1:3-1:6, with medium changes every 2–3 days. Stable lines were selected with puromycin (selection concentration 4.0 µg/mL); antibiotic resistance = puromycin. Cells were cryopreserved in freezing medium (60% basal medium + 30% FBS + 10% DMSO) and stored in liquid nitrogen. Quality control: bacterial, fungal and mycoplasma tests were negative.

Figure S11. Information and validation of *APOE*-KO mice.

A. Strategy of Genotyping.

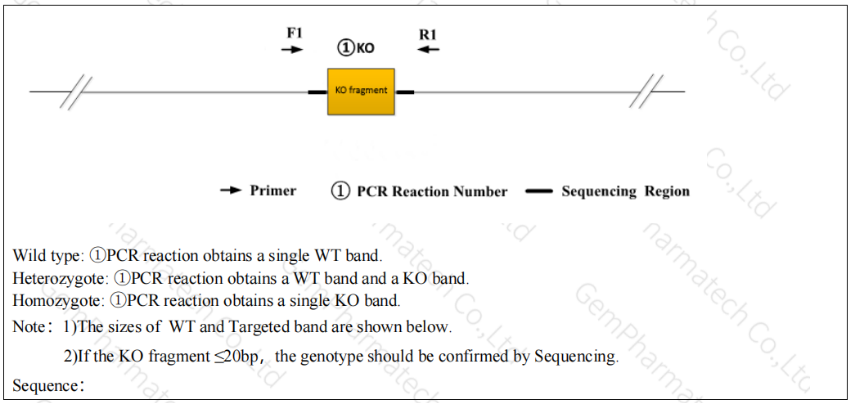

APOE-KO mice were obtained from GemPharmatech Co., Ltd. Primer details are as follows:

Primer Name: T001458-F1; Sequence: TGCCTAGTCTCGGCTCTGAACTAC. Primer Name: T001458-R1; Sequence: CAACCTGGGCTACACACTAATTGAG. Band Size: WT:428bp, KO: 346bp.

B. Representative gel image (reference provided by GemPharmatech Co., Ltd.).

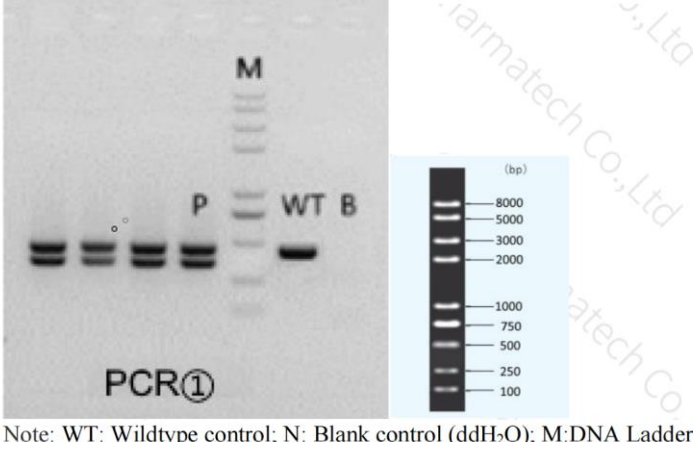

C. Validation of *Apoe* genotyping by PCR: genotyping gel (left) and DNA marker ladder (right). In the genotyping gel, lanes 1–6 correspond to *APOE*-KO mice, and lane 7 corresponds to a wild-type mouse.

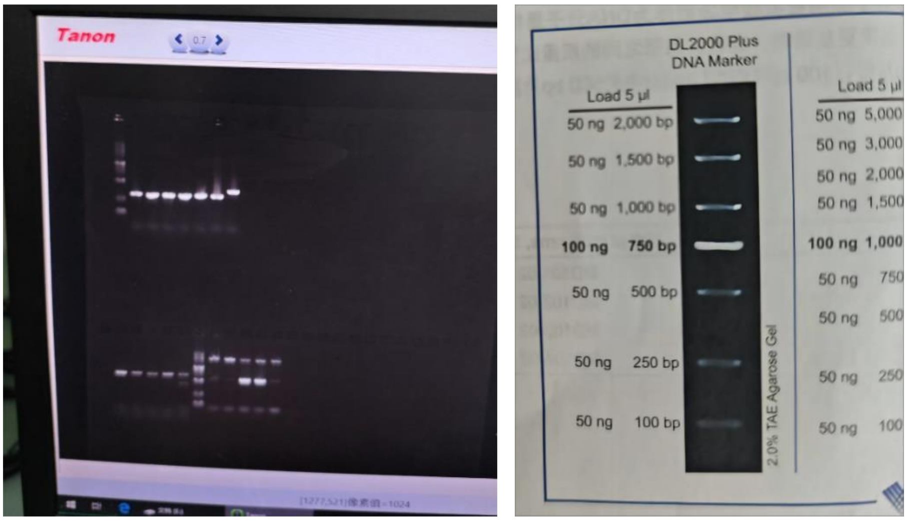

Figure S12. APOE protein expression in the hippocampus of *APOE*-KO, WT, and microglia-AAV-*APOE*-injected mice.

Expression of APOE in the hippocampus of *APOE*-KO, WT, and microglia-AAV-*APOE* mice. Upper: Results of Western blot analysis display the levels of APOE and loading control (β-actin) in hippocampal tissues. Lower: Quantification of the relative expression of APOE/β-actin according to the Western blot data. The data are shown as the Mean ± SD. Statistical significance was determined by one-way ANOVA followed by Tukey's HSD test. A significant effect of group on APOE expression was observed ($F_{(2,15)} = 111.49$, $p < 1 \times 10^{-8}$, $\eta^2 = 0.937$), indicating that genotype/condition explained 93.7 % of the total variance. All pairwise differences were statistically significant, confirming stepwise increases in APOE expression from *APOE*-KO to WT to WT+AAV- *APOE*+KA.

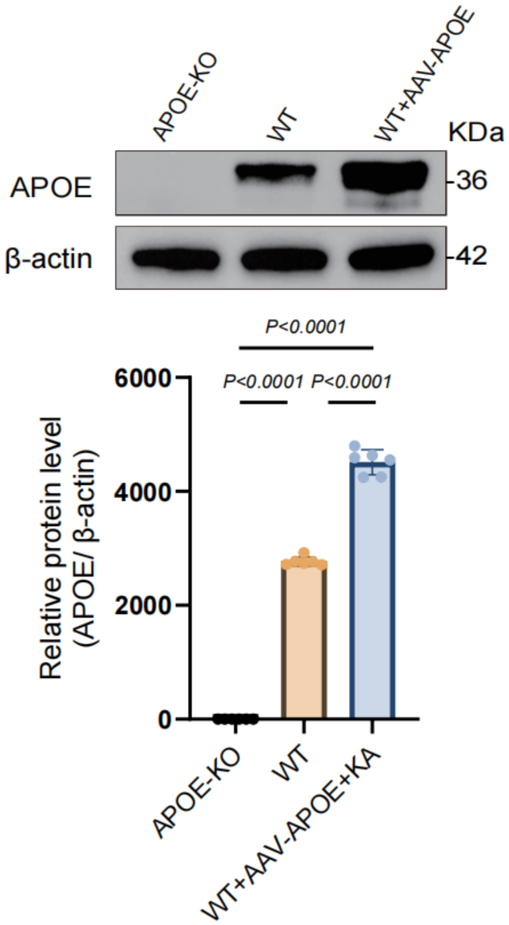

Figure S13. Immunofluorescence validation of microglia-targeted AAV overexpressing *APOE* infection.

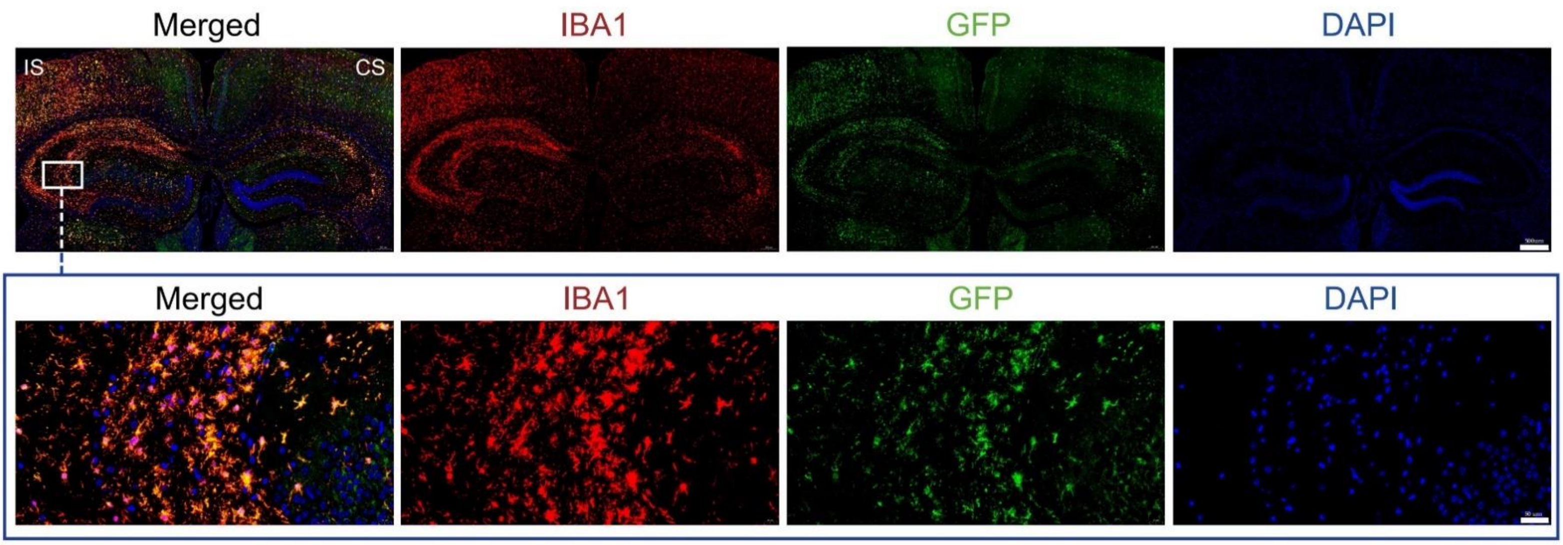

Figure S13 shows the infection efficiency and specificity of the microglia-targeted AAV by immunofluorescence. Top: Low-magnification coronal images of the hippocampus showing GFP reporter expression (green) and the microglia marker IBA1 (red); nuclei are counterstained with DAPI (blue). The ipsilateral side (IS) and contralateral side (CS) are indicated. Bottom: Higher-magnification views of the boxed region in the top panels. The merged high-power image (left) demonstrates extensive colocalization of GFP signal with IBA1-positive microglia (resulting yellow/orange signal in the merge); single-channel panels for IBA1 (red), GFP (green) and DAPI (blue) are shown to the right. Scale bars: 500 µm (top panels) and 50 µm (bottom panels). These representative images confirm efficient and specific targeting of microglia by the AAV-*APOE* construct in the hippocampus.
